# Pulsatile corticoid therapy reduces interictal epileptic activity burden in children with genetic drug‐resistant epilepsy

**DOI:** 10.1002/epi4.12947

**Published:** 2024-06-03

**Authors:** Katharina Schiller, John Thomas, Tamir Avigdor, Daniel Mansilla, Aline Kortas, Gabriele Unterholzner, Markus Rauchenzauner, Birgit Frauscher

**Affiliations:** ^1^ Analytical Neurophysiology Lab, Department of Neurology and Neurosurgery Montreal Neurological Hospital and Institute Montreal Quebec Canada; ^2^ Department of Neurology Children's Hospital Kaufbeuren Kaufbeuren Bavaria Germany; ^3^ Department of Peadiatrics Medical University Innsbruck Innsbruck Austria; ^4^ Department of Neurology Duke University Medical Center Durham North Carolina USA; ^5^ Department of Biomedical Engineering Duke Pratt School of Engineering Durham North Carolina USA

**Keywords:** children, dexamethasone, EEG, epilepsy, interictal activity, sleep spindle

## Abstract

**Objective:**

Corticosteroids and adrenocorticotropic hormone (ACTH) are the therapy of choice to treat infantile spasms. However, systematic studies about their use in other types of childhood epilepsies remain rare and ACTH can have serious side effects. This study compares the interictal epileptic activity (IEA) burden (% of electroencephalography (EEG) time with IEDs) in children with genetic drug‐resistant epilepsy before and after a standardized treatment with pulsatile corticoid therapy (PCT).

**Methods:**

Children with drug‐resistant epilepsy underwent a standardized protocol for PCT with cycles of high‐dose dexamethasone (20 mg/m^2^ body surface) intravenously. Patients were hospitalized for 3 days per PCT cycle and EEGs were obtained before initiation of treatment (baseline) and during the hospitalization around the time of every second cycle. EEG recordings during sleep and wakefulness were obtained. IEA burden was compared before and after PCT. Secondary outcome measures included the sleep spindle rate, the seizure frequency and subjective evaluation in a standardized interview.

**Results:**

In the cohort of 24 children (10 female, 6.2 ± 3.4 years), IEA burden was lower in the EEG after PCT compared to the baseline (baseline: 5.4% [0.7–97.3] vs. after PCT: 1.5% [0–96.9], *p* = 0.001, *d* = −0.41). Sleep physiology expressed by sleep spindles improved after PCT with enhanced fast spindle rates (0.8/min [0–2.2] vs. 1.5/min [0.2–3.4], *p* = 0.045, *d* = 0.36). Seizure frequency was decreased in 17 of the 24 patients (70.8%) with one patient achieving seizure freedom. The majority of patients improved in quality of life (79.2%), and sleep (81.3%). No serious adverse effects were documented.

**Significance:**

This study systematically assessed the effect of PCT in children with genetic / suspected genetic drug‐resistant epilepsy. PCT was found to not only reduce the IEA burden but also increase sleep spindle rates, which are important for cognitive functioning.

**Plain Language Summary:**

In this study, children with a form of epilepsy, which is resistant against antiseizure medication, received a systematic treatment with corticosteroids over multiple cycles in the hospital. It was found that not only the epileptic activity was reduced but also the sleep of the patients was improved after the treatment. These findings could provide the basis for extending the use of corticosteroids in children with epilepsy.

**Key points**

- Corticosteroids and adrenocorticotropic hormone are the therapy of choice to treat infantile spasms. However, their use in other types of childhood epilepsies remains largely unstudied.
- We assessed if a standardized protocol of high‐dose dexamethasone given intravenously reduces the interictal epileptic activity burden in children with genetic / suspected genetic drug‐resistant epilepsy.
- Pulsatile corticoid therapy did not only lead to a reduction of the interictal epileptic activity burden but also increase in sleep spindles important for cognitive functioning.
- Our findings could lay the groundwork for extending the use of pulsatile corticoid therapy in current clinical practice in children with epilepsy.

## 1 INTRODUCTION

Drug‐resistant epilepsy affects about a third of children with epilepsy. Despite its clinical relevance, treatment for drug‐resistant epilepsy remains challenging.^1^ Since the first attempt in the 1950s, corticosteroids, either as adrenocorticotropic hormone (ACTH) or as synthetic preparations of steroids have been used widely in clinical practice to treat infantile spasms and were found to be effective.^2^, ^3^, ^4^, ^5^, ^6^ In patients with West Syndrome, a type of epileptic encephalopathy characterized by infantile spasms, intramuscular ACTH and oral corticosteroids result in seizure reduction up to complete seizure freedom and improvement of electroencephalogram (EEG) findings defined by the disappearance of hypsarrhythmia.^7^, ^8^, ^9^ In a meta‐analysis of van den Munckhof et al.,^10^ steroids led to an improvement in epileptic encephalopathy with electrical status epilepticus in sleep.

According to two systematic reviews, studies evaluating the treatment of corticosteroids in epilepsy types other than infantile spasms are rare.^11^, ^12^ There is only one single double‐blind, crossover clinical trial from 1982 administering ACTH in four children with epilepsy other than epileptic spasms (psychomotor, myoclonic, akinetic, tonic–clonic seizures) highlighting the need for additional comprehensive studies on a larger scale.^11^, ^12^ In the described study, two of the four children with drug‐resistant epilepsy showed an improvement on the only outcome measure of seizure frequency, one patient showed no improvement and one patient dropped out.^13^ The lack of systematic studies was further underlined by a recent review about steroids in childhood epilepsy describing very heterogeneous findings about the effect of steroids in mixed pediatric and adult cohorts.^14^

Few observational studies evaluating intramuscular ACTH and oral steroids in different epilepsy childhood syndromes revealed a positive effect on seizure frequency and subjective evaluation of EEG recordings.^15^, ^16^, ^17^, ^18^, ^19^ However, ACTH may have serious adverse effects such as hypercortisolism, cardiac hypertrophy and electrolyte abnormalities.^20^ Further, ACTH was shown to be associated with brain atrophy.^21^ We therefore propose a standardized protocol of pulsatile cortisone therapy (PCT) using a block design of intervals with hospitalization and high‐dose intravenous dexamethasone. In contrast to oral steroids, the bioavailability of dexamethasone applied through PCT is enhanced and patients can be monitored during the treatment. The dosage of orally administered steroids varied greatly between different studies, as did the duration of administration and choice of steroids.^22^ According to a recent review, there are heterogeneous treatment protocols due to a lack of consensus.^14^ To the best of our knowledge, a systematic prospective study investigating the efficacy of PCT in children with genetic drug‐resistant epilepsy is lacking. In particular, an objective investigation of EEG changes in addition to changes in seizure frequency during PCT is needed to evaluate its clinical use in children with epilepsy. It remains unknown whether PCT could also have beneficial effects on sleep physiology such as sleep spindles. Sleep spindles are sleep transients with a duration of 0.5–3 s and a frequency of 10–16 Hz. They represent the hallmark of N2 sleep and are attributed an important role for memory and learning.^23^ Our group recently found that a local reduction of sleep spindles in patients with focal epilepsy was negatively correlated with cognitive functioning.^24^ Therefore, the objective of this study is to evaluate a standardized treatment of PCT in children with drug‐resistant epilepsy by assessing the interictal epileptic activity burden (IEA, % of EEG time burdened with IEDs) before and after PCT. Secondary endpoints were the improvement of sleep physiology as approximated by the rates of sleep spindles, seizure frequency, and subjective rating of quality of life and sleep between the two time points. We hypothesized that PCT results not only in a reduction of IEDs in the EEG but also in an increase in sleep spindles and a decrease in seizure frequency paralleled by an improvement in everyday functioning.

## 2 MATERIALS AND METHODS

**FIGURE 1:**
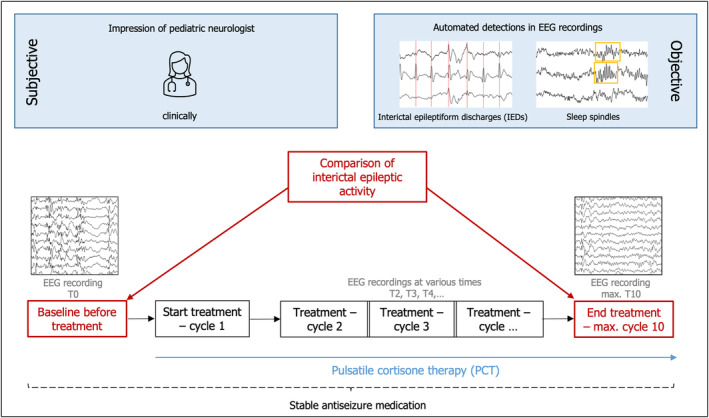
Study design: the IEA burden was compared before and after PCT using the baseline EEG and the EEG after PCT. Additionally, the spindle detections as an objective marker for sleep physiology were compared before and after PCT. Further, the impression of the pediatric neurologist was assessed at the end of treatment to evaluate the efficacy and tolerability of PCT.

For this retrospective study, we included all consecutive pediatric patients with genetic or suspected genetic epilepsy with no whole genome and exome analysis who received the inhouse standardized protocol of PCT administered intravenously in the Children's Hospital Kaufbeuren between 10/2014 and 06/2021. Drug‐resistant epilepsy is defined as failure of two adequately chosen and used antiepileptic drugs to achieve seizure freedom.^25^ Patients receiving PCT were carefully selected by a pediatric neurologist and the treatment was indicated in patients with a significant deterioration in the epileptic seizure burden compared to baseline or cognitive deterioration paralleled with high epileptic activity burden in the EEG, and absence of surgical candidacy. The study was approved by the Ethics board of the Hospital Group Ostallgaeu‐Kaufbeuren (20212). The study design is presented in (Figure 1). Antiseizure medication was kept stable throughout PCT in all patients. No patient received ketogenic diet or vagus nerve stimulation therapy during the time of PCT.

### 2.1 Dexamethasone treatment

Dexamethasone was administered intravenously over 3 days with a dose of 20 mg/m^2^ body surface according to Haberlandt et al.^22^ Patients were hospitalized for 3 days per PCT cycle and EEGs were obtained during the hospitalization around the time of every second cycle depending on the assessment of the pediatric neurologist. Intervals between PCT cycles were 2 to 4 weeks.

### 2.2 EEG

EEG recordings were obtained after sleep deprivation in 21 children in the morning and during wakefulness in three children in the afternoon. Recordings were performed with the Nihon‐Kohden system (Tokyo, Japan). Nineteen electrodes were placed according to the 10–20 system. The mean duration of the EEG recording was 22.8 ± 4.1 min (range: 19.5–35.3). Most children underwent an EEG recording approximately every second cycle. To evaluate the main outcome, we compared the EEG before the start of the PCT as the baseline (difference between baseline EEG and start of PCT: 12.1 ± 17.4 days) and the EEG obtained at the last cycle of PCT. Patients received an average of 7.9 ± 2.4 PCT cycles (range: 2–14), with a maximum of 10 PCT cycles per treatment. One patient (#17) showed a relapse in the EEG after cycle 4 and therefore received a total of 14 cycles.

### 2.3 Sleep scoring

Sleep was scored in 30‐s epochs in a bipolar montage according to the American Academy of Sleep Medicine criteria.^26^ Twenty‐two patients had non‐rapid eye movement (NREM) sleep in both recordings (baseline and at the end of PCT) and three patients had only wakefulness. Stages Wake, N1, N2 and N3 were present in the recordings, and duration of NREM sleep was 15.3 ± 7.1 min (range: 0–26) in all EEGs. In six patients, discrimination between NREM sleep stages was not possible due to abundant epileptic activity at baseline and in four after PCT. Therefore, sleep scoring in these patients resulted in categorization of epochs into either Wake or NREM sleep.

### 2.4 Automated approach for IED detection

An automated approach based on deep learning was applied to perform IED marking in all available EEG recordings and to have an objective comparison between the baseline and the end of PCT. Deep learning approaches for automated IED detection from scalp EEG were shown to yield promising results in focal and generalized epilepsy.^27^, ^28^, ^29^ To train the detector, a minimum of 10 IEDs and a minimum of 10 s of background segments were visually marked in the baseline EEG by a board‐certified neurophysiologist. In three patients, epileptic bursts were marked additionally with a duration marker throughout the EEG recording.

We applied a previously validated convolutional neural network (CNN)‐based architecture for automated IEDs detection.^28^ IEDs and background were considered as a 500‐ms waveform. Channel‐level IED and background annotations from the global annotations were extracted by applying k‐medoids clustering with Dynamic Time Warping as the distance measure.^30^ The detector was then trained on the annotated IEDs at a channel level. To evaluate the data with the CNN IEDs detector, we applied a Leave‐One‐Patient‐Out cross‐validation.

The IED detection was performed at the global level. The output from the 19 channels was combined by applying the maximum of the prediction values. The optimum threshold for IED detection was determined for each patient independently using a semi‐automated approach. The threshold was determined based on the annotated IEDs and background from the individual EEGs with a minimum of 75% sensitivity. The detector was then applied to the EEGs using a sliding window of 500 ms with an overlap of 75% with the previous segment. We performed a visual analysis of the detected IEDs. In case of under‐/over‐detections, the threshold was decreased or increased until a satisfactory number of IEDs were detected. Using these optimized thresholds for each EEG, IEDs were detected.

After applying the trained system for IED detections on the EEG after PCT, a single threshold identical to the threshold at the baseline EEG was used in 15 patients. In 9 patients, after a visual cross‐check by the neurophysiologist, the threshold was lowered to counteract under‐detections. Finally, we achieved a mean sensitivity of 80.2 ± 7.2% for a mean false positive rate of 0.24 ± 0.32 and area under the curve of 0.995 ± 0.01, which is similar to the current literature.^28^, ^29^

### 2.5 IED features

IEA burden was determined as the % duration of the total EEG duration burdened with IEDs. Here we considered each IED be to 250 ms in duration. In three patient EEGs where bursts of IEDs were annotated, the duration of bursts were added to the duration of IEDs. To enable a fair comparison of IEA burden, we selected the vigilance state that was the most prominent in both EEGs (baseline vs. after PCT). In cases where multiple vigilance states were available in the EEG, preference was given to recordings obtained during sleep due to the higher IED rates that are typically observed in NREM sleep.^31^

Further, we compared the number of channels involved in IED events as well as the proportion of IEDs with preceding gamma activity in the 30–70 Hz frequency band.^32^, ^33^ The detections were visually validated by a board‐certified neurophysiologist. The IED gamma proportion is reported as the percentage of IEDs with gamma activity. We analyzed IED‐gamma in patients with segments of deeper sleep (N2 + N3) in both recordings (baseline and after PCT, *n* = 11).

Finally, an independent epileptologist, blinded toward automated IED markings and the clinical findings, was asked to report whether he observed an improvement, no improvement, or worsening regarding the change in epileptic activity between the baseline EEG and EEG at the end of PCT.

### 2.6 Sleep spindle detections

To evaluate sleep physiology, we applied an automatic detection of sleep spindles with a validated spindle detector^34^ in the common average montage in all 19 channels exclusively in N2 sleep. The criteria were set to a frequency of 10–16 Hz and a duration of 0.5–3 s using a custom script written in MATLAB 2020b implemented in our lab. The detector has been previously used in patients with epilepsy.^35^ A smoothing moving window of 300 ms and 80th percentile thresholds was applied. Further, spindles were categorized as fast spindles (12–16 Hz) or slow spindles (10–12 Hz). Detections at the time of an IED were excluded due to potential misdetections. We set a minimum duration criterion of 1 min of N2 sleep in the baseline EEG and EEG after PCT which was fulfilled by 12 patients. We included two additional patients where sleep scoring was not possible at baseline but at the end of PCT when N2 sleep was present. Therefore, assessment of spindle rates was performed in 14 patients. For each participant, detections were visually cross‐checked by a board‐certified neurophysiologist.

### 2.7 Subjective rating from parents, pediatric neurologist and adverse effects

The pediatric neurologist assessed seizure frequency at baseline and at the end of PCT based on the parents' report and a patient diary. A decrease of 50% or more in seizure frequency after PCT was considered as an improvement by the pediatric neurologist. If seizures were subclinical, information regarding seizure improvement was not applicable. Parents of study participants were asked at the end of the PCT in a standardized interview whether they observed an improvement in their child regarding quality of life and sleep. Parents were asked to rank each measure with improvement or no improvement. If the answer was not applicable (e.g. seizure count in case of subclinical seizures) or parents did not have an opinion, a third option of information not applicable/no opinion was chosen.

Patients underwent a clinical examination by a pediatrician on admission to the unit for each cycle of PCT. Occurrence of adverse effects due to PCT was registered in the patient's file. Because hyperglycemia is a potential adverse effect during PCT,^17^, ^36^ blood glucose measurement was performed in addition to a complete blood count on admission to the unit for each cycle of PCT and monitored if necessary.

## 3 EVALUATION AND STATISTICS

Data were tested for normal distribution using the Kolmogorov–Smirnov test. Due to non‐normal distribution of the IEA burden (%), spindle rates (min), number of channels involved in IED events, and IED gamma proportion (%), data are reported as median and range (median [range]). IEA burden, spindle rates and number of channels involved in IED events were compared using the Wilcoxon signed rank test for paired samples. Effect sizes are reported using Cliff's delta. Statistical analyses were performed using MATLAB 2020a with a *p*‐value <0.05 considered to indicate statistical significance. Multiple comparisons were corrected using the false discovery rate procedure.

## 4 RESULTS

Twenty‐four children (10 female; mean age ± SD, 6.2 ± 3.4 years; age range: 0.5–13.7 years) were included in the final analysis (Table 1). All patients had a likely genetic etiology, of which seven patients had the following genetic variants: FBXO11 (#4), KCNT1 (#9), SLC35A2 (#10), SLC6A1 (#12), triple15ql (#19), STX1B (#20), partial trisomy 14 and 20 (#21). According to the classification of the International League against Epilepsy (ILAE),^37^ 11 patients had focal epilepsy syndromes, eight patients had generalized syndromes, five patients had syndromes with developmental and epileptic encephalopathy with progressive neurological deterioration. MRI was normal in all patients except one patient, for whom dysgenesis of the corpus callosum was observed. Clinical information of the study participants is presented in Supplementary Material S1.

**TABLE 1:** Clinical characteristics of study participants and EEG parameters.

Total number of study patients (*n*)	24
Sex (*n*, female/male)	10/14
Age (mean ± SD)	6.2 ± 3.4 years
Type of epilepsy (*n*)	
Focal epilepsy syndromes	11
Generalized epilepsy syndromes	8
Syndromes with developmental epileptic encephalopathy	5
Epilepsy syndrome (*n*)	
Myoclonic epilepsy in infancy	6
Self‐limited epilepsy with centro‐temporal spikes	6
Developmental epileptic encephalopathy	5
Epilepsy with myoclonic absences	3
Self‐limited epilepsy with autonomic seizures	2
Epilepsy with eyelid myoclonia	1
Juvenile absence epilepsy	1
Number of PCT cycles (mean ± SD)	7.9 ± 2.4
EEG recording during NREM sleep/wakefulness (*n*)	21/3
Duration of EEG recordings (mean ± SD)	22.8 ± 4.1 min

Abbreviations: EEG, electroencephalography; NREM, non rapid eye movement; PCT, pulsatile corticoid therapy; SD, standard deviation.

### 4.1 Interictal epileptic activity burden

**FIGURE 2:**
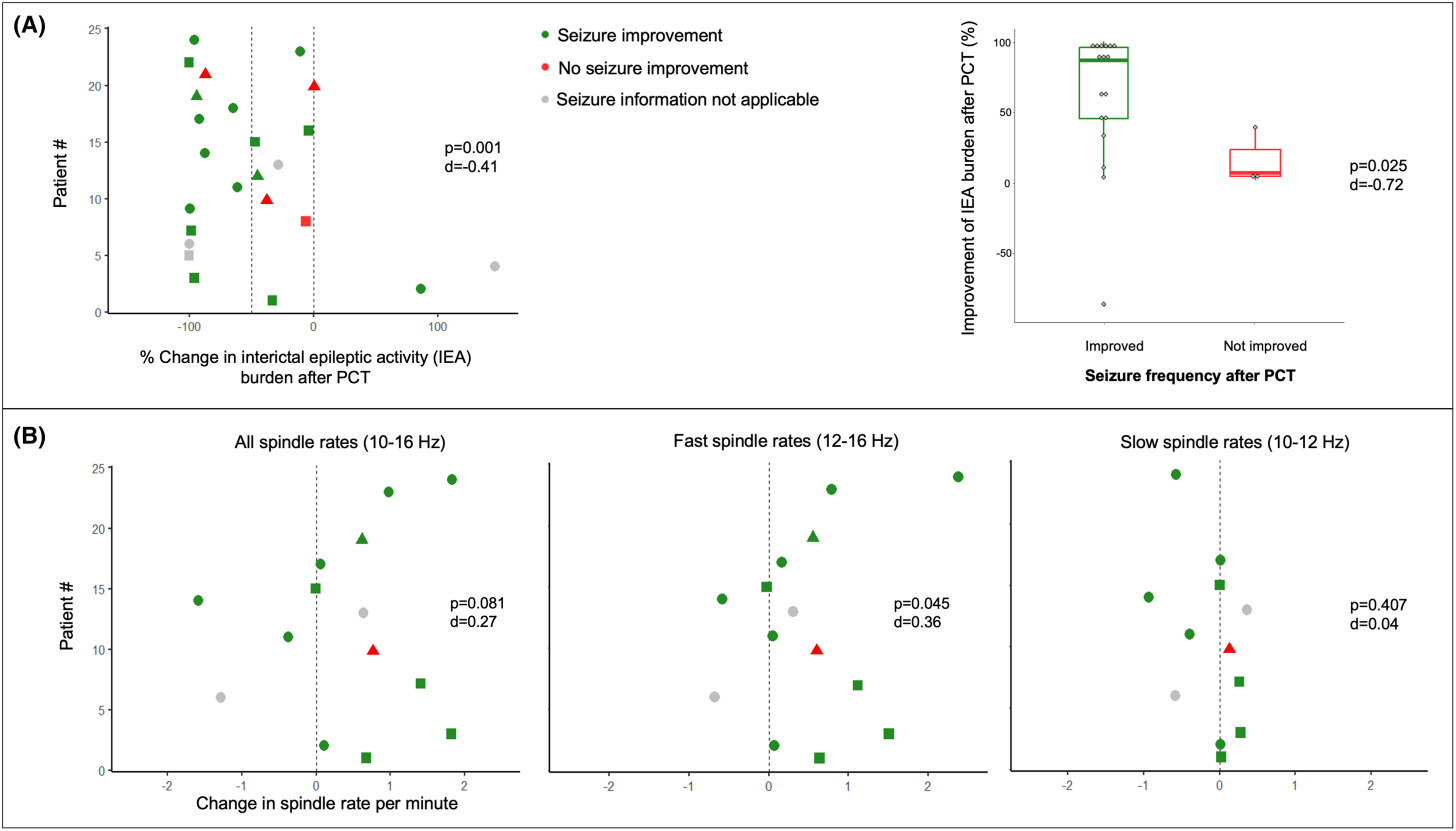
(A) IEA burden presented as percentage change is significantly reduced after PCT. Percentage change below zero corresponds to a reduction in IEA burden and percentage change above zero to an increase in IEA burden. Patients with improvement of seizure frequency showed significantly higher reduction in IEA burden. (B) Fast spindle rates presented in change in rate per minute in N2 are significantly increased after PCT, whereas there was no difference in all and slow spindle rates. Each square/dot/triangle represents one patient. Generalized epilepsy patients are represented by a square, focal epilepsy by a dot, developmental and epileptic encephalopathy syndromes with progressive neurological deterioration by a triangle.

**FIGURE 3:**
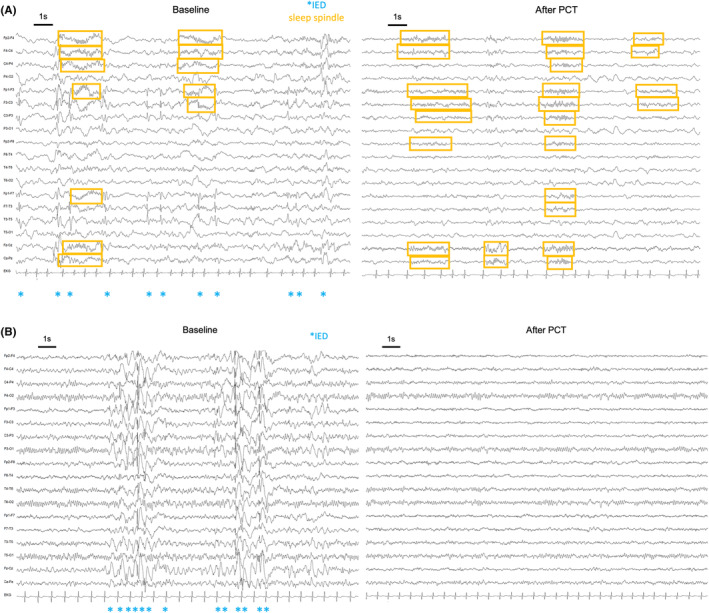
(A) EEG recording with 19 channels in a bipolar montage (20 μV/mm, LF: 0.5, HF: 70, 20s/page) in sleep stage N2 at baseline and after 8 cycles of PCT in sleep stage N2 in patient #24. Sleep spindles are indicated in orange boxes and IEDs as blue stars. (B) EEG recording with 19 channels in a bipolar montage (20 μV/mm, LF: 0.5, HF: 70, 20s/page) in wakefulness at baseline and after 6 cycles of PCT in sleep stage N2 in patient #22. IEDs are indicated as blue stars. The patient became free from their IEA burden after PCT.

**FIGURE 4:**
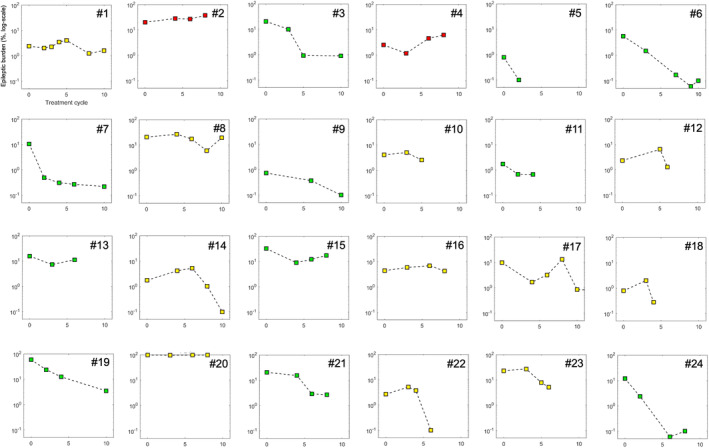
Change in IEA burden over the different treatment cycles. Patients who showed a continuous decline in IEA burden are colored in green (*n* = 11), patients with a decline after an initial increase in yellow (*n* = 11) and patients with worsening in red (*n* = 2). We changed the IEA burden in this depiction to a log scale to be able to apply the same scale on all patients for better comparison.

IEA burden (% of EEG time burdened with IEDs) was compared during Wake (in three patients), N1 (in nine patients), and N2 + N3 (in 12 patients). The burden was significantly lower in the EEG after PCT compared to the baseline EEG (baseline: 5.4% [0.7–97.3] vs. after PCT: 1.5% [0–96.9], *p* = 0.001, *d* = −0.41) (Figure 2A). Four patients became free from IEA burden (no IEDs or bursts) after PCT. Patients with improved seizure frequency showed significantly higher reduction in IEA burden than patients who did not show a decrease in seizures (improvement of IEA burden: 86.7% [−86.5.7–100.0] vs. 5.9% [0.4–38.1], *p* = 0.025, *d* = −0.72 Figure 2A). An EEG example of the baseline recording and the recording after PCT is presented in Figure 3A and Figure 3B. Examples of EEG recordings before and after PCT of all 24 patients are available in Supplementary Material S2. The change in the IEA burden over different treatment cycles is presented in Figure 4. The number of channels involved in IEDs per patient was not significantly different between baseline and after PCT (95th percentile: before PCT: 5 [1–14] vs. after PCT: 5 [1–17], *p* = 0.33, *d* = 0.05). The IED gamma proportion was not significantly different before and after PCT (*n* = 11, before PCT: 14.30% [7–53] vs. after PCT: 28.60 [0–60], *p* = 0.37, *d* = .20).

The ratings of the independent epileptologist revealed an improvement of 19 EEG recordings after PCT and no improvement in five patients. The five patients rated with no improvement by the epileptologist also showed no improvement (#2, #4) or a difference around zero (#8, #16, #20) according to the automated IED detections. Therefore, we observed a high concordance between the automated detections and the ratings of the independent epileptologist.

### 4.2 Sleep spindle rates

**FIGURE 5:**
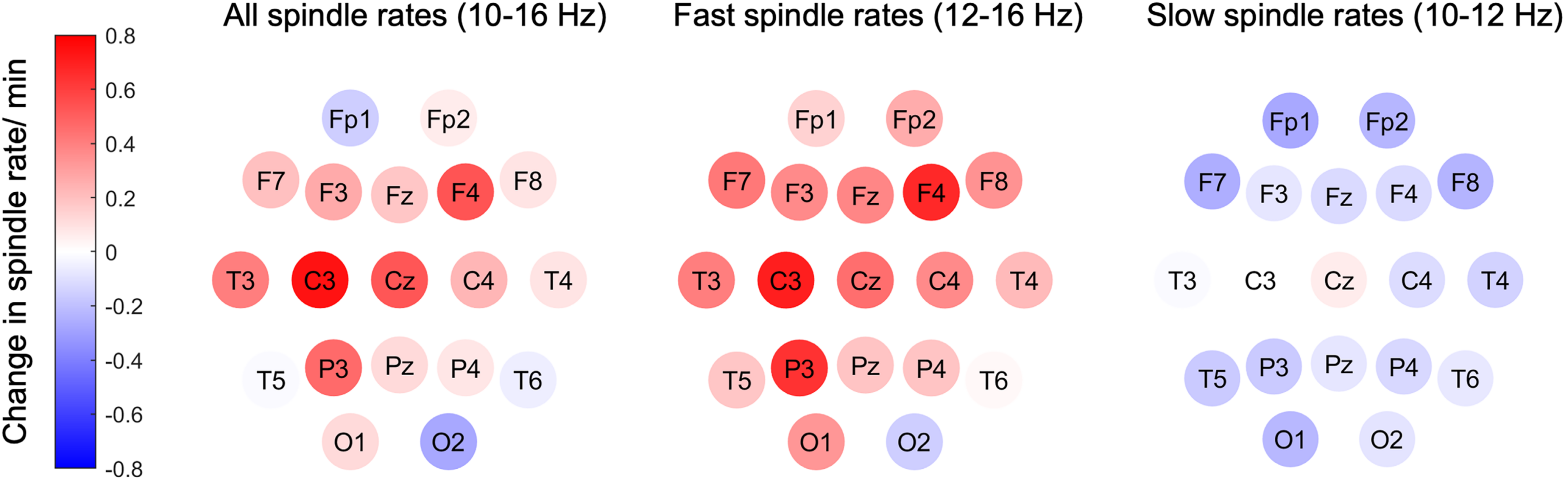
The difference in spindle rates before and after PCT averaged over all spindle detections in 14 patients. Positive values indicate an increase in spindle rates after PCT. All spindles (10–16 Hz), fast spindles (12–16 Hz) and slow spindles (10–12 Hz) show a fronto‐centro‐parietal maximum. Fast spindles were significantly increased after PCT.

Spindle rates in 8.7 ± 4.2 min of N2 sleep were analyzed in 14 patients who showed at least one minute of N2 sleep in both EEG recordings. Spindle rates were slightly increased in the EEG after PCT compared to the baseline EEG (baseline: 1.0/min [0–3.2] vs. after PCT: 1.6/min [0.2–3.9], *p* = 0.081, *d* = 0.27) (Figure 2B). Fast spindle rates (Figure 2B) were significantly increased in the EEG after PCT compared to the baseline EEG (baseline: 0.8/min [0–2.2] vs. after PCT: 1.5/min [0.2–3.4], *p* = 0.045, *d* = 0.36) in contrast to slow spindle rates which did not differ between baseline and after PCT (baseline: 0.3/min [0–1.2] vs. after PCT: 0.3/min [0.1–0.5], *p* = 0.407, *d* = 0.04) (Figure 2B). The difference in spindle distributions before and after PCT is depicted in Figure 5.

### 4.3 Subjective measurements

Of all 24 patients, the change in seizure frequency was not available in four patients as seizure manifestations were not accompanied by objective clinical symptoms. Of the remaining 20 patients, 17 patients (85.0%) showed improvement in the seizure frequency and three patients (15.0%) showed no improvement. One patient became seizure‐free. Quality of life (19 of 24 children, 79.2%) and sleep (13 of 16 children, 81.3%) were as well improved after PCT. No patient was reported to show an increase in seizure frequency.

### 4.4 Adverse effects

No serious adverse effects such as serious infections or weight gain were registered during the PCT. Nineteen patients (79.2%) reported light adverse effects such as sleepiness (*n* = 10), mood swings (*n* = 9), stomach‐ache (*n* = 3), restlessness (*n* = 2), reduced appetite (*n* = 2), bloating (*n* = 3), diarrhea (*n* = 1), increased appetite (*n* = 1), weakness (*n* = 1), fever (*n* = 1), muscle pain (*n* = 1), joint pain (*n* = 1), difficulties to fall asleep (*n* = 1), and microcytic anemia (*n* = 1). Two patients showed hyperglycemia (>140 mg/dL) at admission, although glucose concentration became normalized during the hospitalization.

## 5 DISCUSSION

Drug‐resistant epilepsy accounts for up to 80% of the healthcare burden in epilepsy^38^ and the treatment for pediatric patients with genetic drug‐resistant epilepsy is limited because epilepsy surgery is not an option. Therefore, it is important to study other treatment options to reduce IEA burden and improve daily functioning in this significant group of patients. This is the first study to systematically assess the efficacy of PCT in children with genetic drug‐resistant epilepsy syndromes following a standardized protocol. We found that PCT was an efficacious treatment with no major side effects which were assessed by the clinical team and blood work‐up. More specifically, (i) IEA burden was reduced after PCT, (ii) sleep physiology expressed by sleep spindles was improved, and (iii) seizure frequency was decreased after PCT.

### 5.1 Reduction of IEA burden after PCT

In the vast majority of patients, PCT led to an improvement of activity in the EEG recordings with 11 patients showing a reduction over 50% in the IEA burden and four patients no longer having an IEA burden at the end of the treatment. In previous studies, administration of ACTH^19^, ^39^ in patients with Landau–Kleffner syndrome or oral dexamethasone in children with epileptic encephalopathy^15^ was reported to improve EEG recordings using a subjective rating. In contrast to these studies, we used an objective measure and quantified the IEA burden using an automated deep learning‐based detector. We found a significant decline in epileptic activity after applying the standardized protocol of PCT at the last cycle of treatment. Our objective findings were supported by a high overlap with ratings of an independent epileptologist blinded to all clinical information. The reduction of epileptic activity is relevant for various reasons: IEDs during NREM sleep were not only found to be negatively correlated with sleep spindles^40^ but also found to disrupt sleep, impact cognition and disturb the coupling of physiological sleep oscillations necessary for long‐term memory consolidation.^41^, ^42^, ^43^ The reduction of IEDs may therefore have a positive effect not only on sleep but also on cognitive functions.

### 5.2 Improvement of sleep physiology after PCT

PCT not only had a positive effect on pathophysiology by reducing epileptic seizure burden but also was associated with an improvement in sleep physiology. This is the first study to objectively assess the effects of corticosteroids on sleep physiology expressed by sleep spindles in children with genetic drug‐resistant epilepsy. Sleep spindles are physiological sleep oscillations hallmarking N2 sleep. Fast sleep spindles (12–16 Hz) were enhanced at the end of PCT. This is important as sleep spindles play an important role in cognition and learning^23^ and fast spindles were found to better predict cognition than slow spindles.^44^ PCT may hence not only have the potential to reduce the IEA burden but also to have a positive impact on sleep physiology and cognitive functioning.

### 5.3 Improvement of seizure frequency

Seizure frequency was reduced in two thirds of patients after PCT with one patient becoming seizure‐free. This is in line with studies reporting an improvement in seizure burden using ACTH and oral steroids in children with drug‐resistant epilepsy outside West syndrome^15^, ^16^, ^17^ as well as in the double‐blind clinical trial conducted by Pentella et al. in five patients.^13^ While the assessment of seizure frequency is highly subjective due to its dependence on the reports of parents and patients, the IEA burden in the EEG recordings can be evaluated objectively. Bringing both measures together, we showed a significantly higher reduction in IEA burden in the patient group with improved seizure frequency. However, this will need to be confirmed in future studies by using standardized neuropsychological tests.

Moreover, seizure frequency is negatively correlated with quality of life^45^ and at the end of PCT, 79.2% of children were reported to have an enhanced quality of life which may be related to the reduction in seizure frequency or as well to the improvement of sleep physiology as most patients also showed improved subjective sleep quality. Reported adverse effects were only marginal making PCT well tolerated. It is important to note that the listed adverse effects were only registered during the days of hospitalization and not in everyday life outside the treatment cycle and mostly at the beginning during the first cycles of PCT.

### 5.4 Strengths and limitations

By using an objective approach to quantify the IEA burden and avoid subjectivity in the outcome evaluation, we aimed to pave the way and provide support for clinical treatment with PCT. The reduction of the IEA burden in the EEG and the clinical improvement of patients can be seen as a treatment with high potential for treating children with genetic drug‐resistant epilepsy. Potential confounding of the outcome by antiseizure medication can be ruled out as medication was kept stable throughout PCT and no patient received other non‐pharmacological treatment such as ketogenic diet or neuromodulation during PCT. Nevertheless, there are some limitations to our study. First, the treatment was not given in a randomized and placebo‐controlled clinical trial. However, as the patient group consisted of children with drug‐resistant epilepsy who showed a deterioration in epileptic seizure burden or cognitive decline, administering a placebo for the long duration required for the treatment with PCT would not be ethically responsible. Additionally, the design did not offer the possibility to use a historical control group, as antiseizure medication would not have been kept stable as was done here during PCT treatment. Another limitation could be the short duration of the EEG recordings. Polysomnography recordings would offer the possibility to explore sleep macro‐ and microstructure and epileptic activity in more detail. However, we aimed to strike a balance between the quality of the recording and feasibility due to the burden on the child. Furthermore, follow‐up measurements were not examined in the present study and therefore, the long‐term development as well as the stability of the effect after PCT cannot be discussed. Finally, the reports of parents regarding subjective perceived parameters may be biased and highly subjective.

### 5.5 Conclusion

Using an innovative study design, we systematically evaluated the effect of intravenous dexamethasone in children with different types of drug‐resistant epilepsy. PCT was found to not only reduce the pathophysiology of epilepsy but also improve physiological measurements during sleep that are deemed important for cognitive functioning. This study may lay the groundwork to extend the use of PCT in current clinical practice based on both efficacy and low side effect potential.

## AUTHOR CONTRIBUTIONS

Conception and design of the study: K.S., M.R., B.F.; Acquisition and annotation of data: K.S., J.T., D.M., A.K., G.U., M.R., B.F.; Statistical analysis and interpretation of results: K.S., J.T., T.A., M.R., B.F.; Manuscript preparation: K.S., M.R., B.F.; Manuscript revision and approval: all authors.

## CONFLICT OF INTEREST STATEMENT

None of the authors has any conflict of interest to disclose. We confirm that we have read the Journal’s position on issues involved in ethical publication and affirm that this report is consistent with those guidelines.

## ETHICS STATEMENT

The data are available to qualified investigators upon written request to the corresponding author Markus Rauchenzauner and if in compliance with regulations of the local Institutional Ethics Review Board. This study was approved by the Ethics board of the Hospital Group Ostallgaeu‐Kaufbeuren (20212). As this research was retrospective, informed consent was waived.

## Supporting information


Data S1.

## Data Availability

Data are available to qualified investigators upon written request to the corresponding author Markus Rauchenzauner and if in compliance with regulations of the local Institutional Ethics Review Board.
