# Supplementary material for: Pulsatile corticoid therapy reduces interictal epileptic activity burden in children with genetic drug‐resistant epilepsy
Source: Epilepsia Open. 2024 Jun 3;9(4):1265–76. doi: 10.1002/epi4.12947 (PMC11296103; doi:10.1002/epi4.12947)
Supplement: Supplementary file 1 — Data S1. [file EPI4-9-1265-s001.pdf]

Supplementary Material

Supplementary Material 1. Clinical characteristics of study participants.

ID	Age (y); sex	ILAE Epilepsy syndrome		Aetiology	EEG pathology (before PCT)	MRI	Cycles PCT	ASM/day during PCT
1	4.7; M	Myoclonic epilepsy in Infancy	generalized	genetic	frequent epileptiform anomaly with bifrontal maximum	normal	10	CLB 20mg; ZNS 14,4ml
2	8.4; F	Self-Limited Epilepsy with Centro-Temporal Spikes	focal	genetic	frequent epileptiform anomaly over fronto-central with shifting side preponderance	normal	8	STM 200mg; VPA 600mg
3	2.8; F	Myoclonic epilepsy in Infancy	generalized	genetic	abundant focal epileptiform anomaly over left central >> right central	normal	10	VPA 420mg; LEV 560mg
4	9.8; F	Self-Limited Epilepsy with Centro-Temporal Spikes	focal	genetic (FBXO11)	frequent focal epileptiform disturbance over right centro-temporal	normal	8	LTG 225mg; STM 250mg
5	4.9; M	Myoclonic epilepsy in Infancy	generalized	genetic	occasional diffuse epileptiform anomaly or focal central with shifting preponderance	normal	2	VPA 660mg; LTG 72.5mg; CBZ 6mg
6	8.9; M	Self-Limited Epilepsy with Centro-Temporal Spikes	focal	genetic	abundant focal epileptiform anomaly over centro-temporal left and frequent over centro-temporal right	normal	10	STM 250mg; BRV 100mg
7	4.7; M	Epilepsy with Eyelid Myoclonia	generalized	genetic	abundant focal epileptiform anomaly over vertex	normal	10	VPA 390mg; PRN: DZP 10mg
8	4.9; M	Myoclonic epilepsy in Infancy	generalized	genetic	abundant diffused epileptiform anomaly	normal	10	LEV 1000mg
9	7.6; M	Self-Limited Epilepsy with Autonomic seizures	focal	genetic (KCNT1)	frequent epileptiform disturbance over left occipital	normal	10	LEV 1000mg; OXC 250mg
10	0.5; F	Developmental Epileptic Encephalopathy	DEE/PND	genetic (SLC35A2)	abundant diffuse and multifocal epileptiform anomaly	normal	5	VGB 860mg; PRN: DZP 5mg
11	6.3; M	Epilepsy with Myoclonic Absences	focal	genetic	occasional frontal epileptiform anomaly with shifting preponderance	normal	8	LTG 250mg; ESM 500mg; CLB 12ml

12	1.8; M	Developmental Epileptic Encephalopathy	DEE/PND	genetic (SLC6A1)	frequent epileptiform anomaly over left hemisphere with parieto-centro-temporal maximum	normal	6	-
13	5.9; M	Self-Limited Epilepsy with Centro-Temporal Spikes	focal	genetic	abundant epileptiform anomaly with centro-temporal maximum left >> right	normal	6	STM 100mg
14	9.4; M	Self-Limited Epilepsy with Centro-Temporal Spikes	focal	genetic	occasional epileptiform anomaly over right temporo-occipital	normal	10	STM 300mg; VPA 750mg;
15	8.8; F	Myoclonic Epilepsy in Infancy	generalized	genetic	frequent generalized rhythmic delta wave EEG discharges with superimposed frontal preponderant spikes	normal	8	VPA 750mg; PRN: BM 7,5mg
16	7.2; F	Epilepsy with Myoclonic Absences	generalized	genetic	frequent diffuse and multifocal epileptiform disturbance	normal	8	ESM 500mg; CLB 30mg
17	2.8; M	Self-Limited Epilepsy with Autonomic seizures	focal	genetic	abundant focal epileptiform anomaly over midline and right parieto-occipital area and occasional ill-defined sharp waves left frontal	normal	14	VPA 360mg; STM 75mg
18	13.7; M	Juvenile Absence Epilepsy	focal	genetic	occasional focal epileptiform anomaly with spikes frontopolar bilateral	normal	4	ZNS 200mg; PRN: BM 10mg
19	0.6; F	Developmental Epileptic Encephalopathy	DEE/PND	genetic (triple 15ql)	Abundant generalized and multifocal epileptiform anomalies	normal	10	PB 50mg; VPA 240mg; OXC 288mg; PRN: DZP 5mg
20	3.6; F	Developmental Epileptic Encephalopathy	DEE/PND	genetic (STX1B)	abundant multifocal epileptiform anomaly	normal	8	CLZ 1mg; VPA 420mg; LEV 900mg
21	13.1; M	Developmental Epileptic Encephalopathy	DEE/PND	genetic (partial trisomy 14 and 20)	abundant multifocal epileptiform anomaly with lefthemispheric preponderance	Dysgenesis of the corpus callosum,	8	LTG 300mg; CLB 5mg

						Dandy Walker variant		
22	7.8; F	Epilepsy with Myoclonic Absences	generalized	genetic	abundant generalized epileptiform anomaly	normal	6	LEV 1400mg; VPA 900mg
23	5.9; F	Myoclonic Epilepsy in Infancy	focal	genetic	abundant left hemispheric epileptiform anomaly with shifting preponderance	normal	6	VPA 630mg
24	5.6; M	Self-Limited Epilepsy with Centro-Temporal Spikes	focal	genetic	abundant focal epileptiform anomaly over left front-temporal area	normal	8	LEV 800mg; PRN: BM 7,5mg

Abbreviations: BM=Buccal Midazolam, BRV=Brivaracetam, CLB=Clobazam, CZP=Clonazepam, DEE=Developmental and Epileptic Encephalopathy, DZP=Diazepam, ESM=Ethosuximide, F=female, ILAE=International League against Epilepsy, LAC=Lacosamide, LEV=Levetiracetam, LTG=Lamotrigine, LZP=Lorazepam, M=Male, OXC=Oxcarbazepine, PB=Phenobarbital, PCT=Pulsatile Corticoid Therapy, PND=Progressive Neurological Deterioration, PRN=pro re nata, STM=Sulthiame, VGB=Vigabatrin, VPA=valproic acid, ZNS=Zonisamide

***Supplementary Material 2*.** Selection of EEG recordings of all 24 patients before and after PCT.

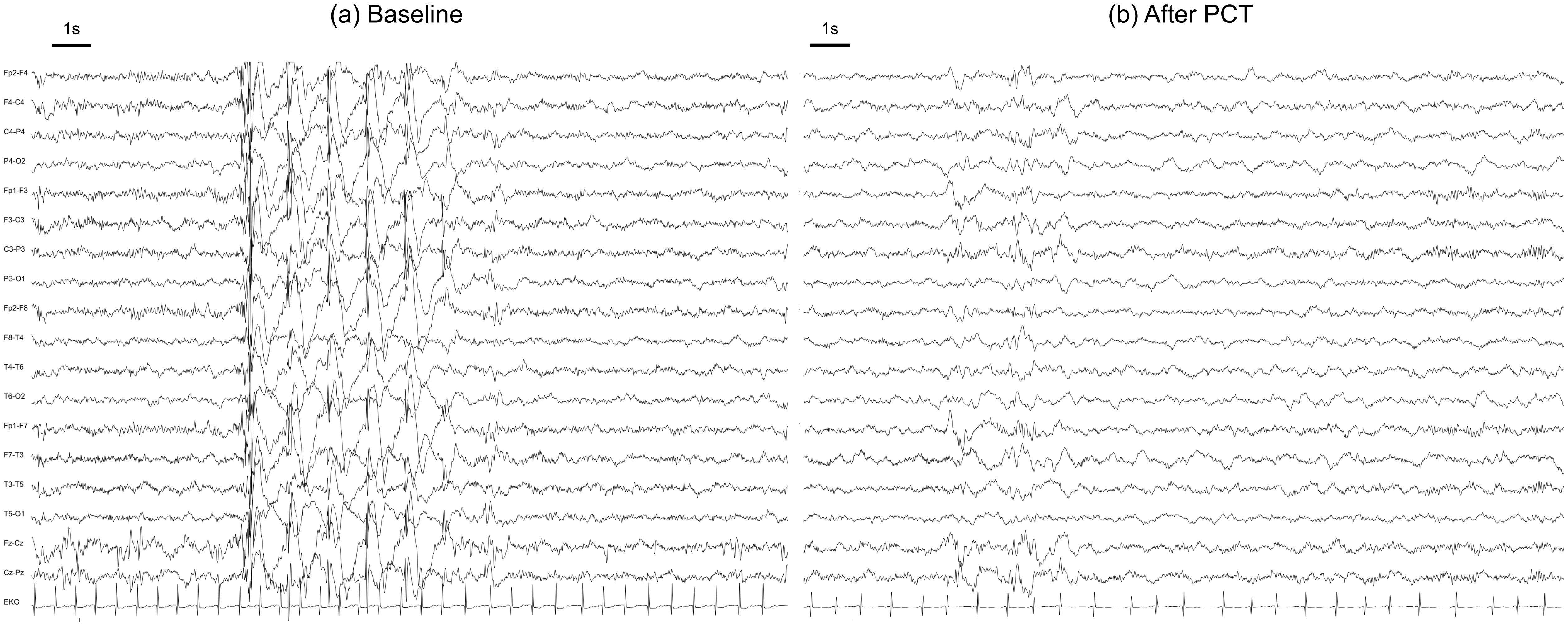

EEG recording with 19 channels in bipolar montage (20μV/mm, LF: 0.5, HF:70, 20s/page) during sleep stage N2 at baseline (a) and after 10 cycles of PCT during sleep stage N2 (b) in patient #1.

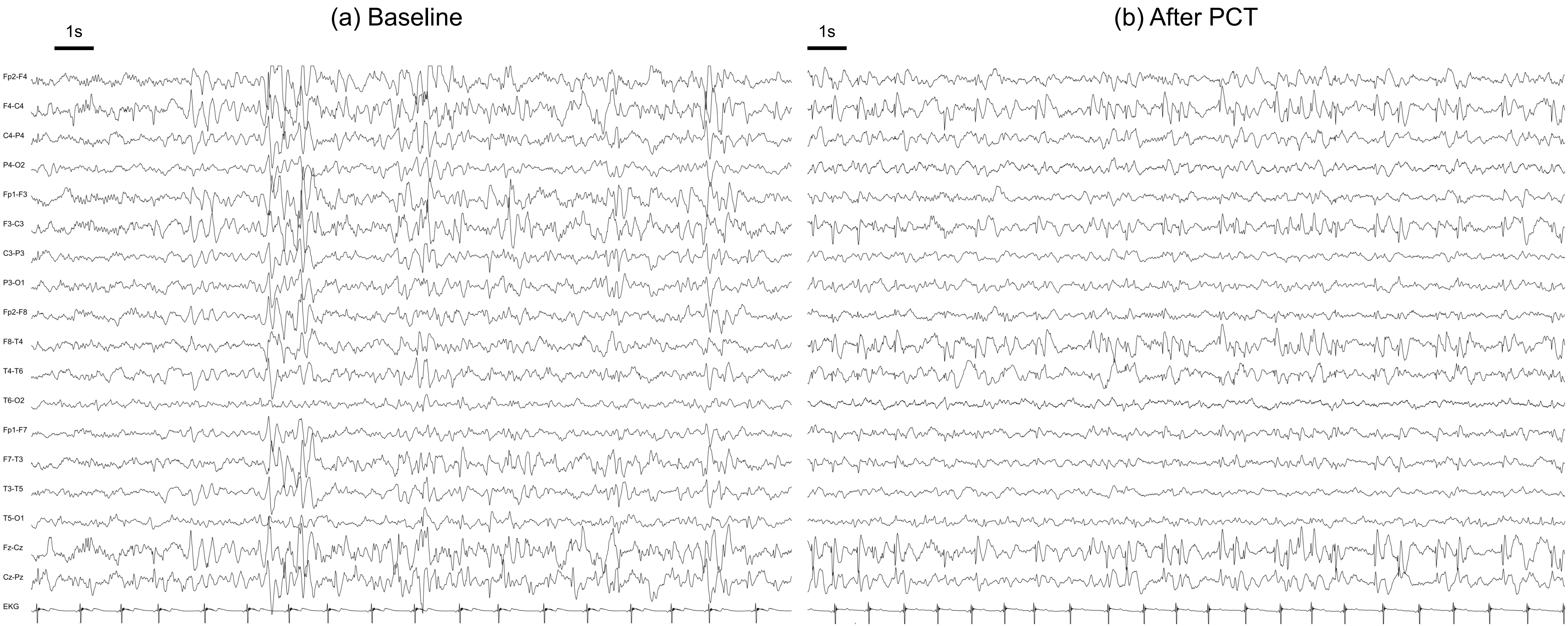

EEG recording with 19 channels in bipolar montage (20μV/mm, LF: 0.5, HF:70, 20s/page) during sleep stage N2 at baseline (a) and after 8 cycles of PCT during sleep stage N2 (b) in patient #2.

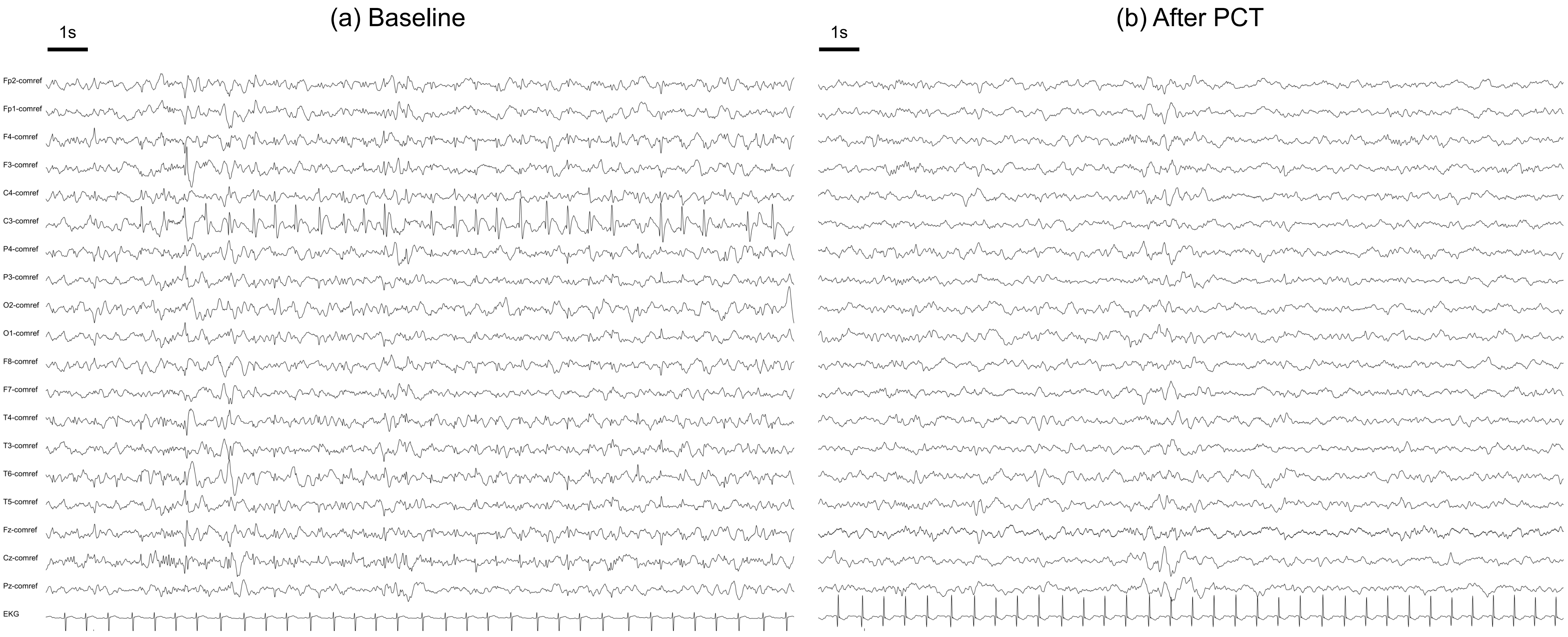

EEG recording with 19 channels in common average montage (20µV/mm, LF: 0.5, HF:35, 20s/page) during sleep stage N2 at baseline (a) and after 10 cycles of PCT during sleep stage N2 (b) in patient #3.

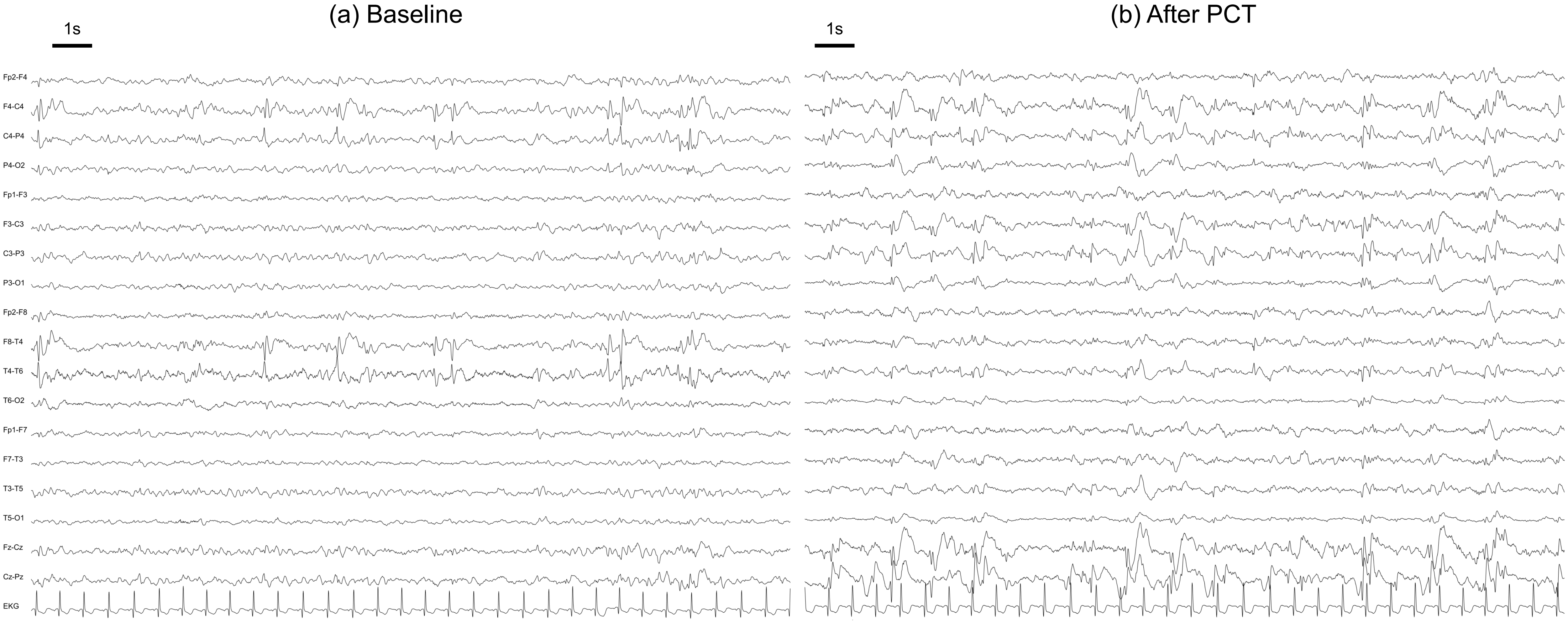

EEG recording with 19 channels in bipolar montage (20μV/mm, LF: 0.5, HF:70, 20s/page) during sleep stage N1 at baseline (a) and after 8 cycles of PCT during sleep stage N1 (b) in patient #4.

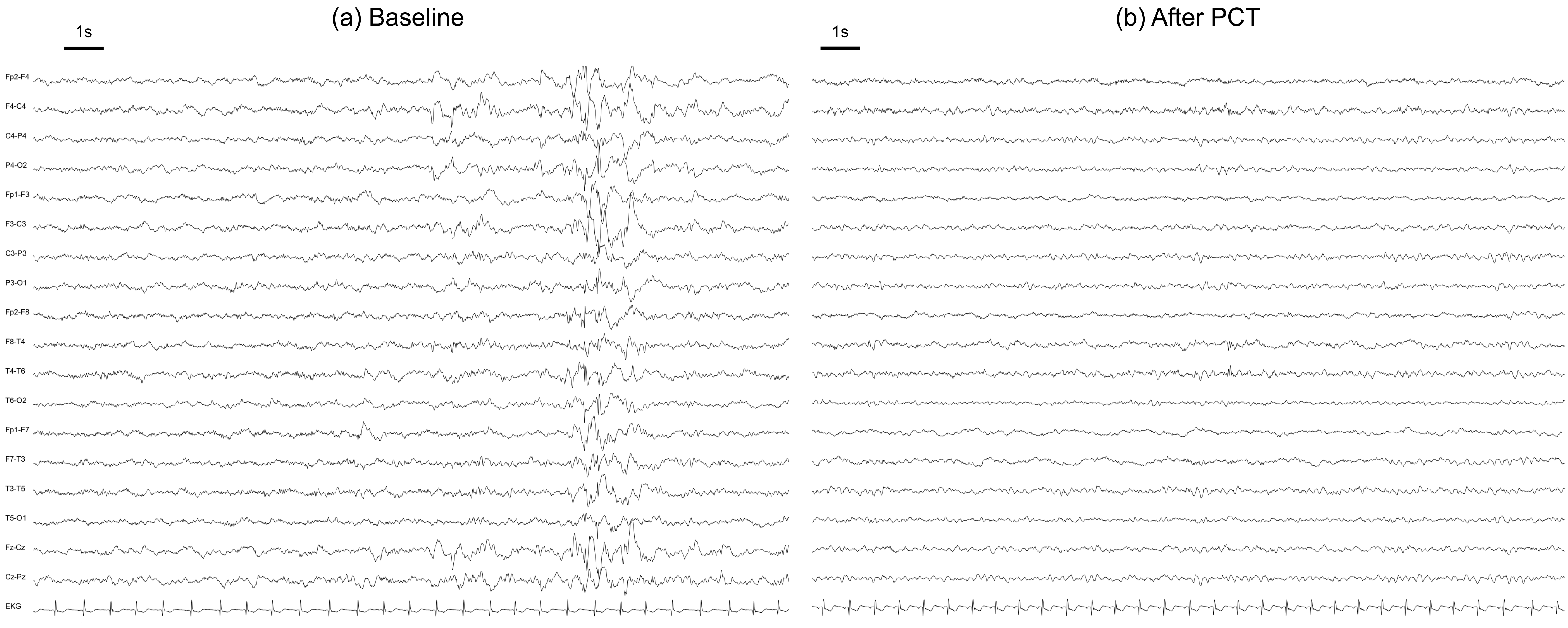

EEG recording with 19 channels in bipolar montage (20μV/mm, LF: 0.5, HF:70, 20s/page) during sleep stage N1 at baseline (a) and after 2 cycles of PCT during sleep stage N1 (b) in patient #5.

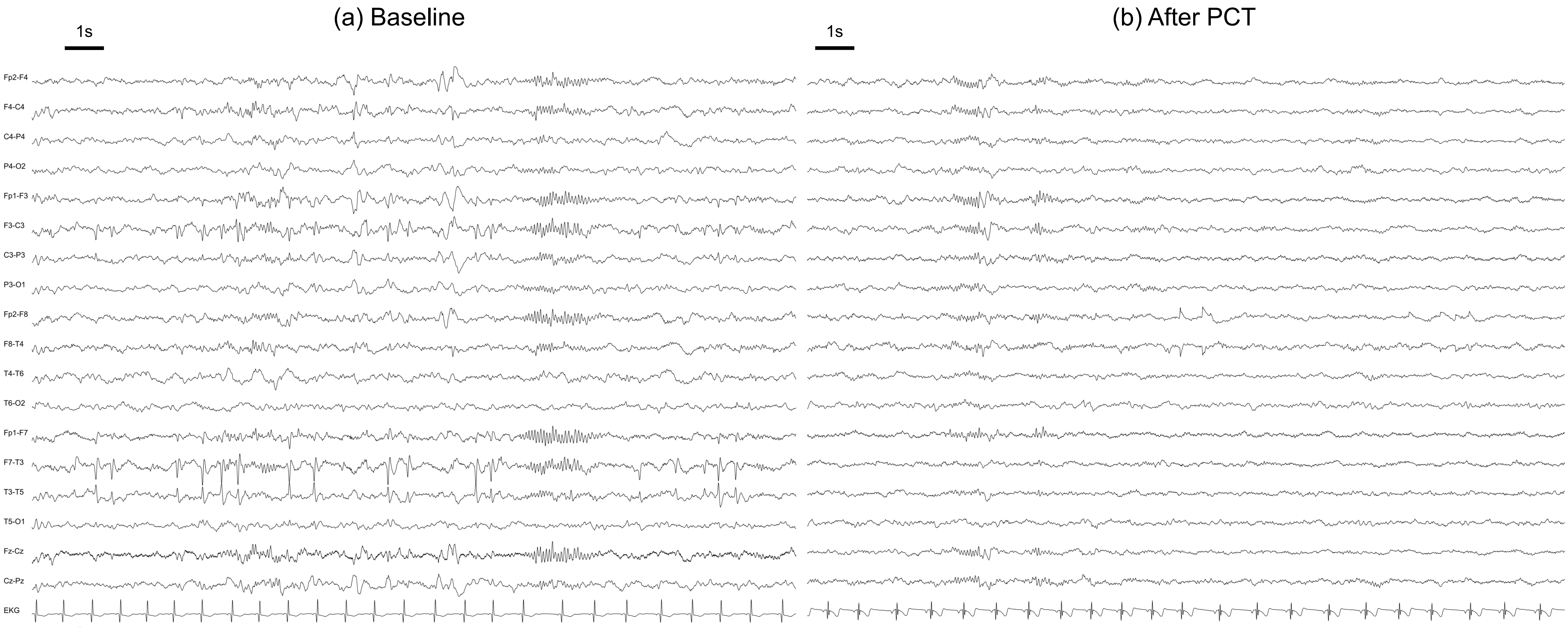

EEG recording with 19 channels in bipolar montage (20μV/mm, LF: 0.5, HF:70, 20s/page) during sleep stage N2 at baseline (a) and after 10 cycles of PCT during sleep stage N2 (b) in patient #6.

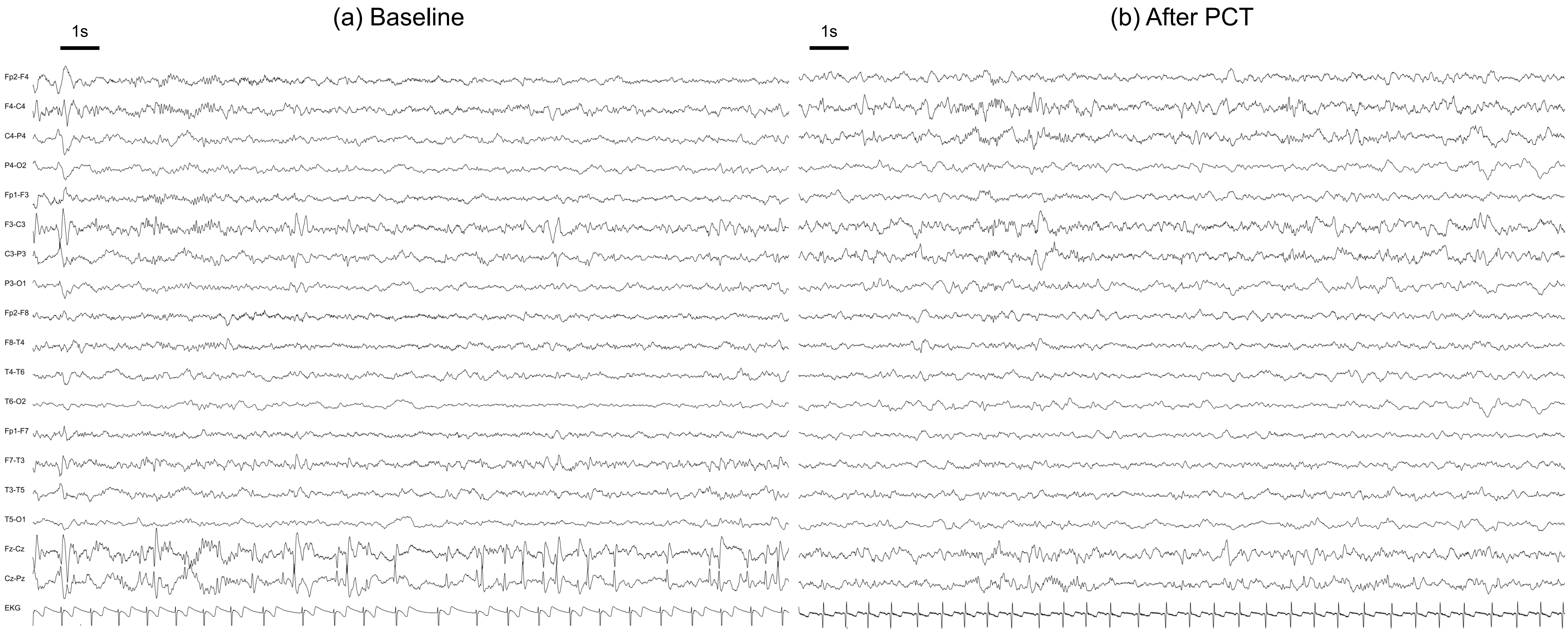

EEG recording with 19 channels in bipolar montage (20µV/mm, LF: 0.5, HF:70, 20s/page) during sleep stage N2 at baseline (a) and after 10 cycles of PCT during sleep stage N2 (b) in patient #7.

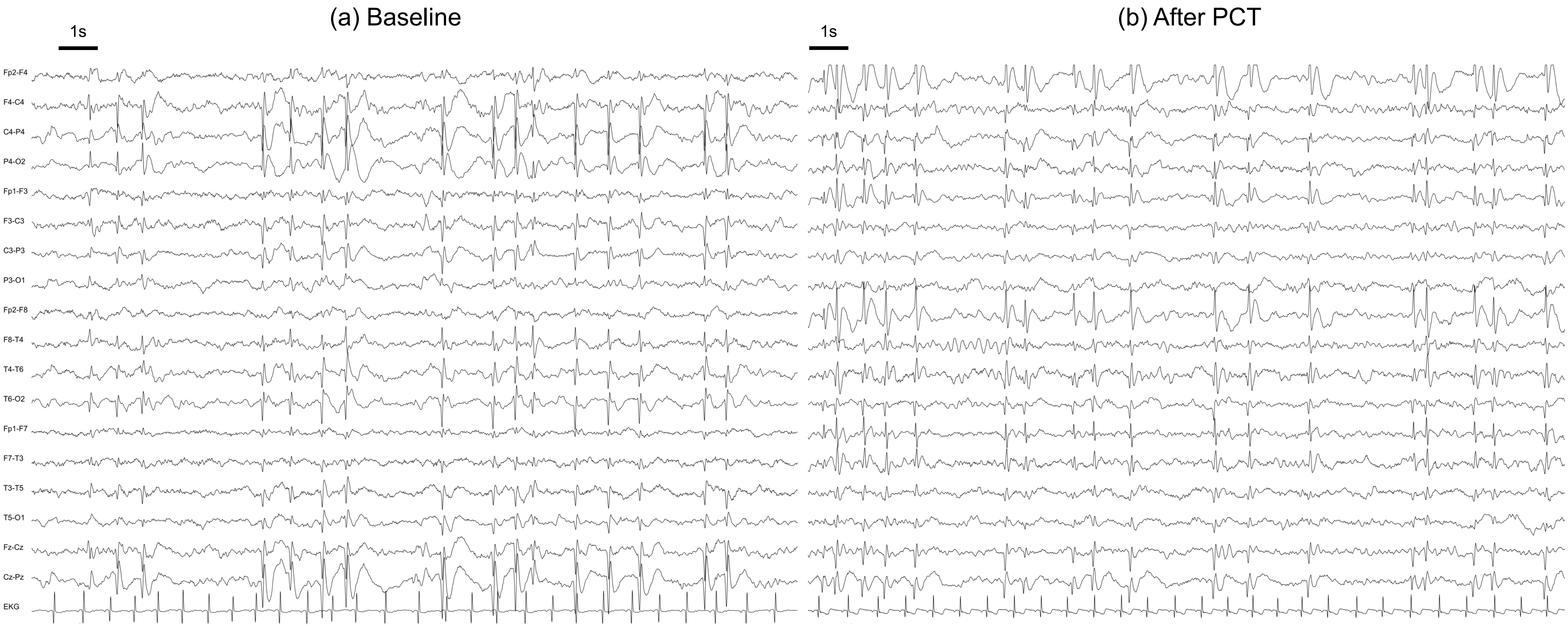

EEG recording with 19 channels in bipolar montage (20μV/mm, LF: 0.5, HF:70, 20s/page) during sleep stage N1 at baseline (a) and after 10 cycles of PCT during sleep stage N1 (b) in patient #8.

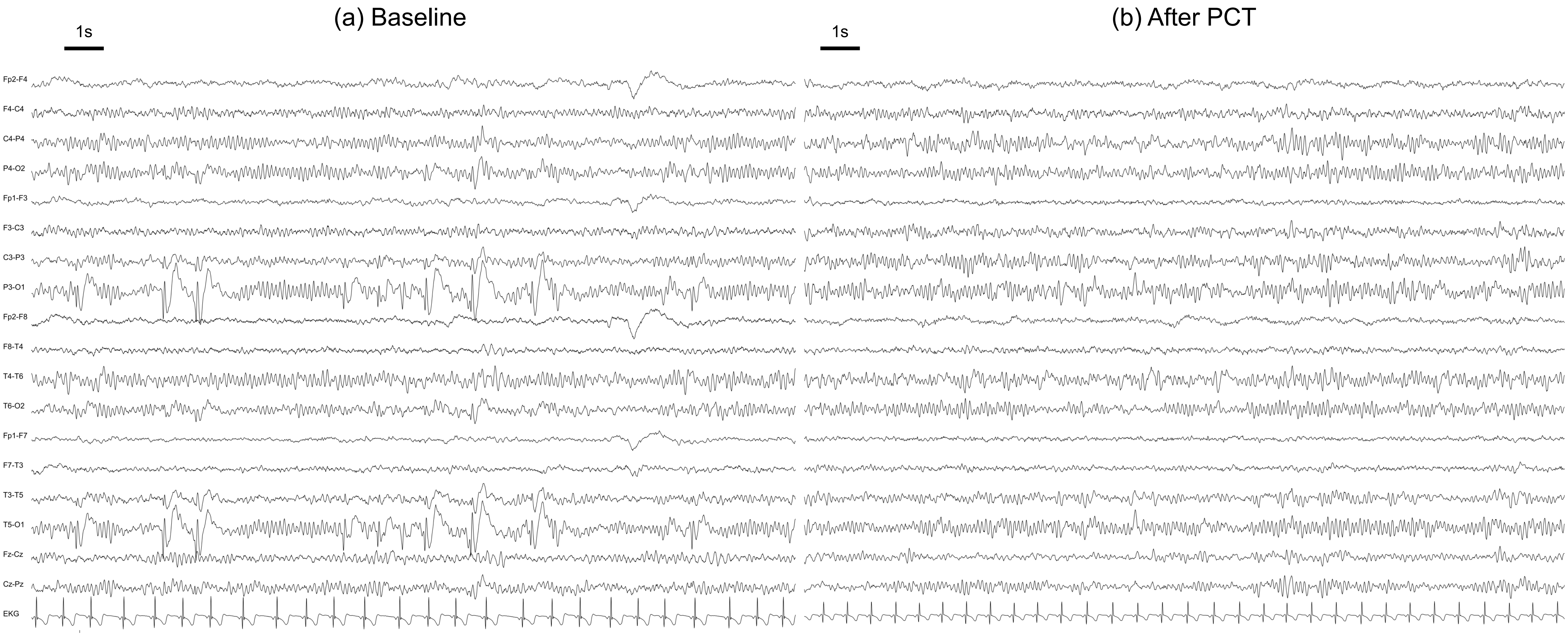

EEG recording with 19 channels in bipolar montage (30µV/mm, LF: 0.5, HF:70, 20s/page) during wakefulness at baseline (a) and after 10 cycles of PCT during wakefulness (b) in patient #9.

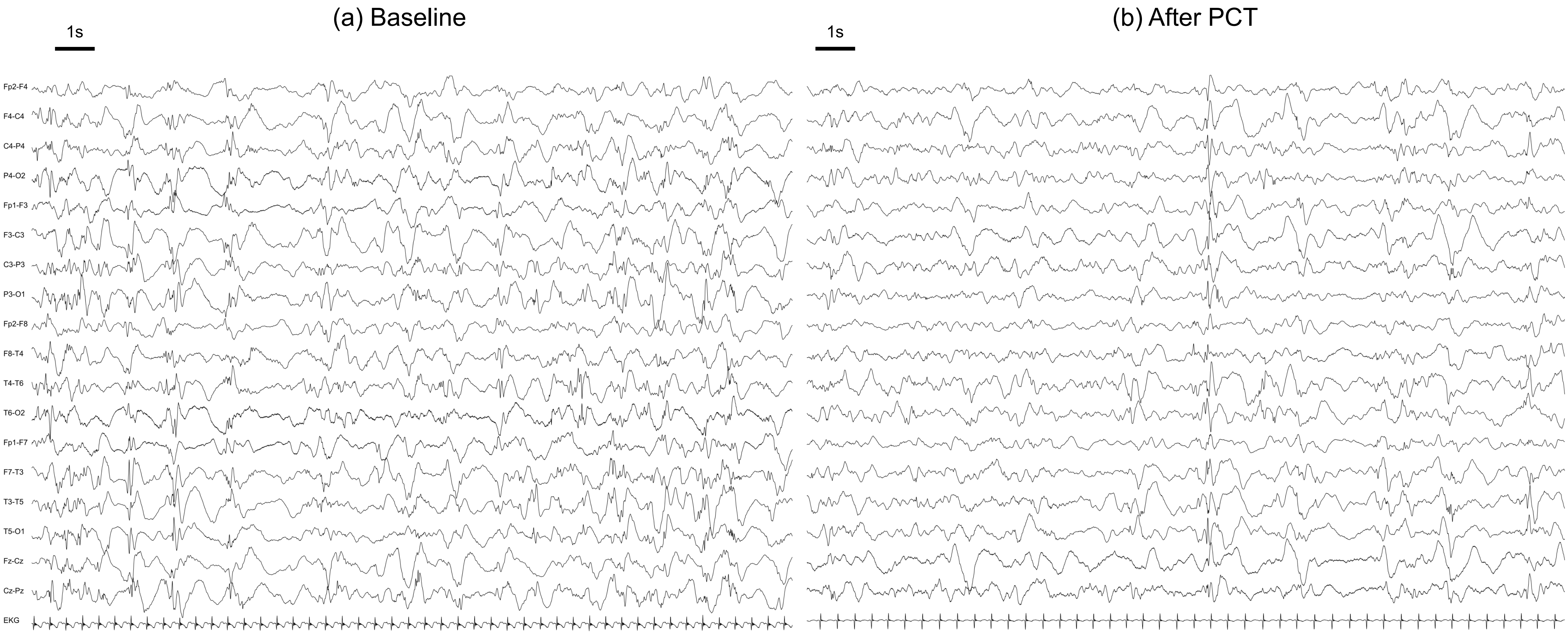

EEG recording with 19 channels in bipolar montage (20µV/mm, LF: 0.5, HF:70, 20s/page) during in sleep stage N3 at baseline (a) and after 5 cycles of PCT in sleep stage N3 (b) during patient #10.

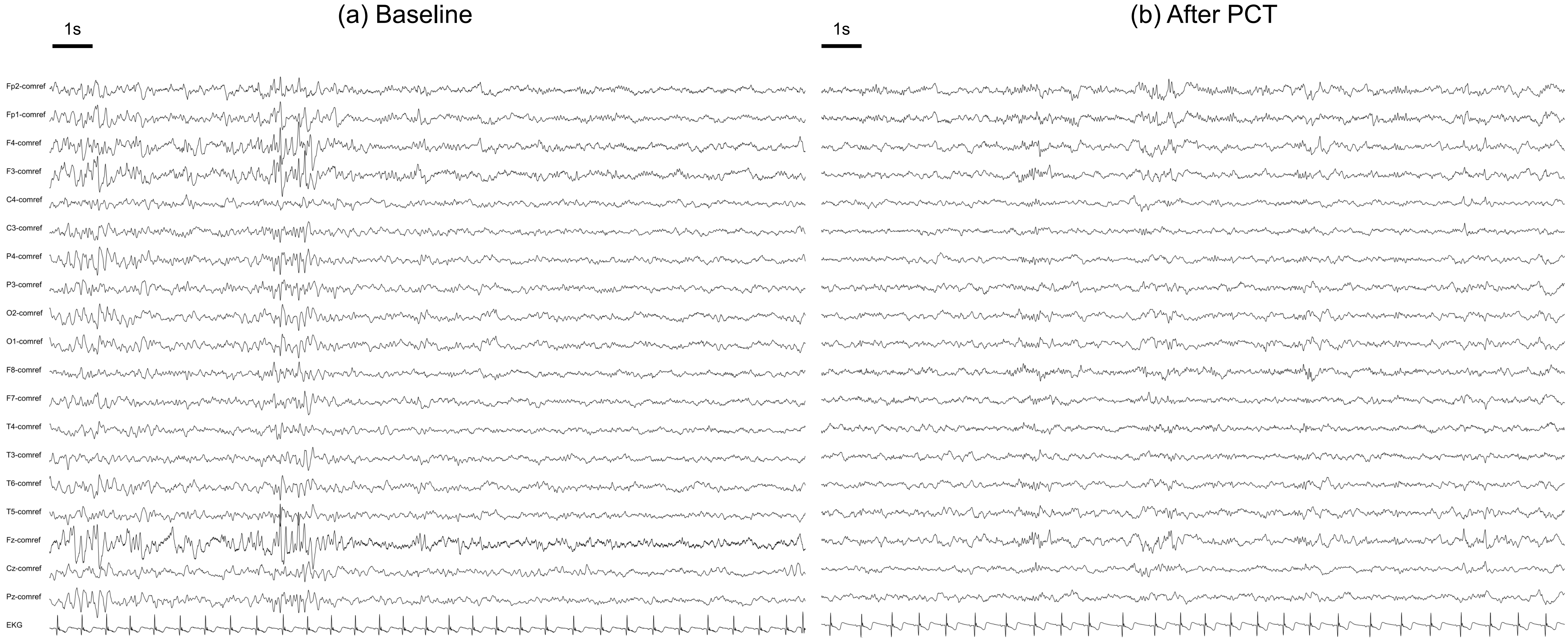

EEG recording with 19 channels in common average montage (20μV/mm, LF: 0.5, HF:70, 20s/page) during sleep stage N2 at baseline (a) and after 8 cycles of PCT during sleep stage N2 (b) in patient #11.

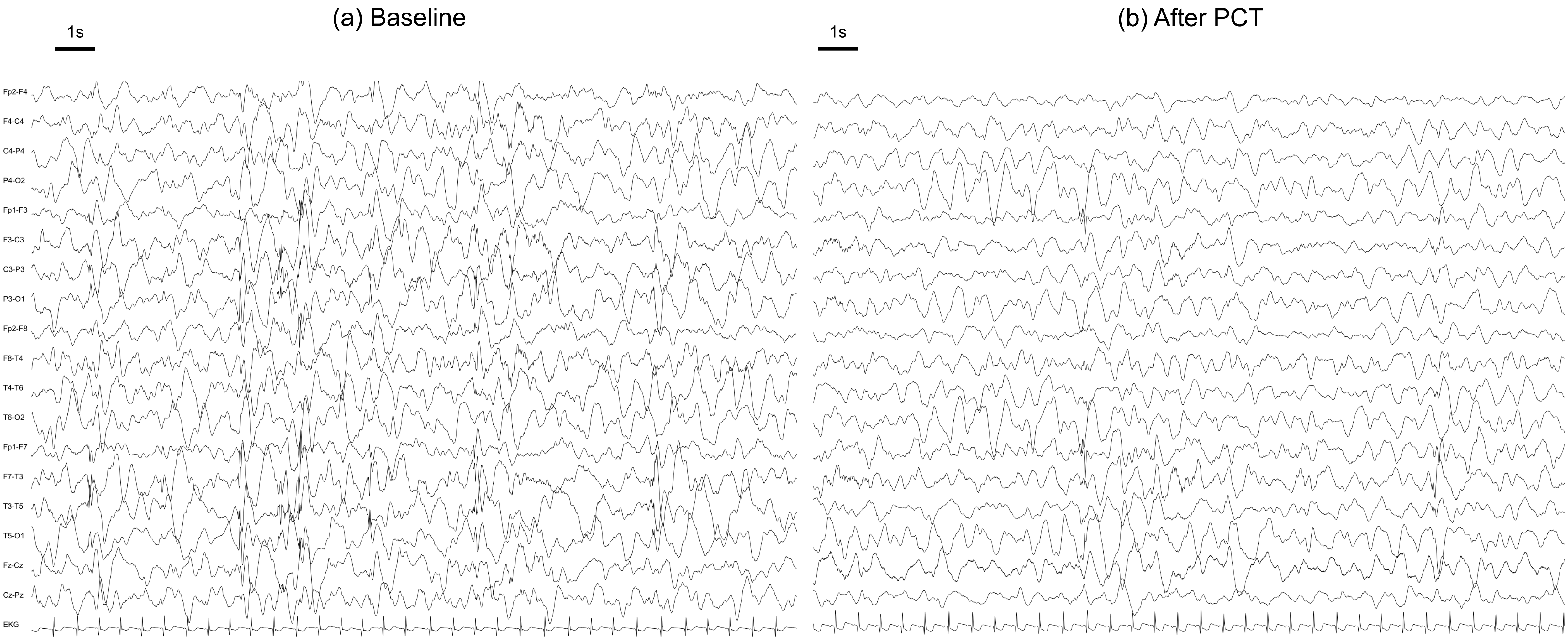

EEG recording with 19 channels in bipolar average montage (20μV/mm, LF: 0.5, HF:70, 20s/page) during sleep stage N3 at baseline (a) and after 6 cycles of PCT during sleep stage N3 (b) in patient #12.

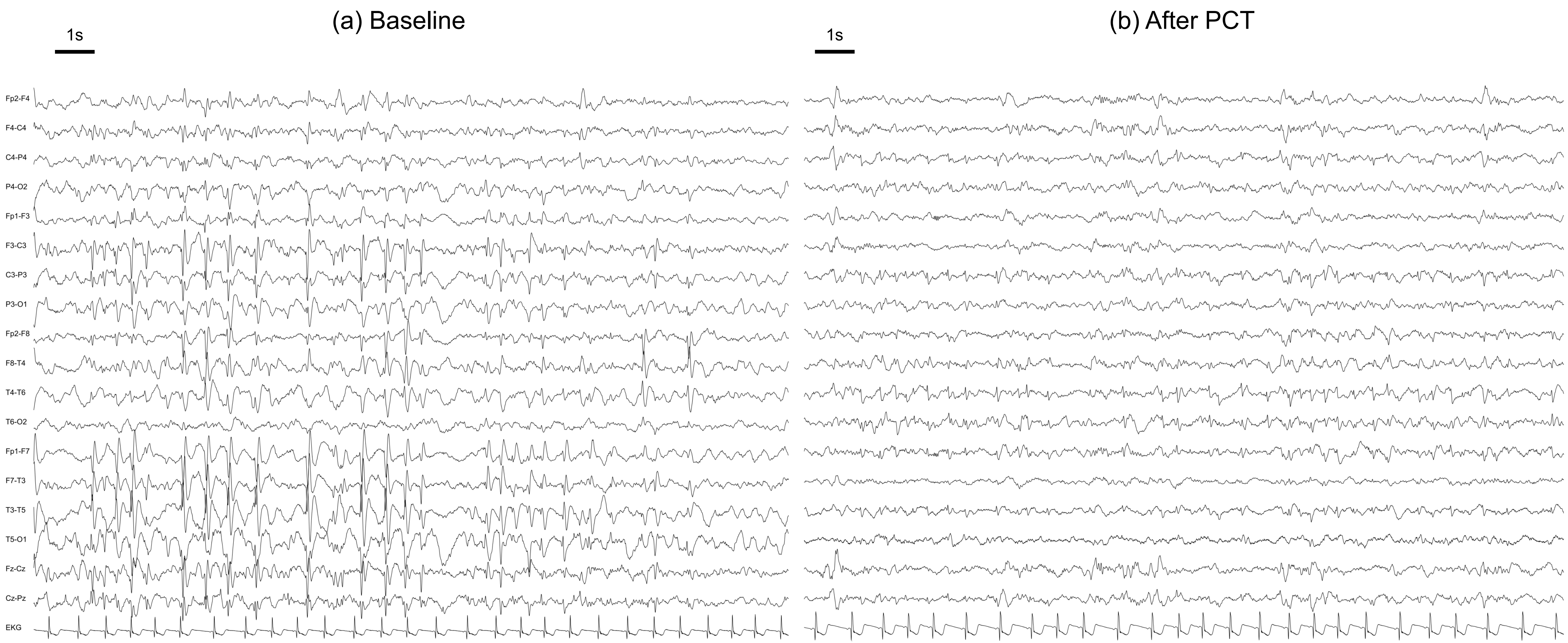

EEG recording with 19 channels in bipolar montage (20µV/mm, LF: 0.5, HF:70, 20s/page) during sleep stage N1 at baseline (a) and after 6 cycles of PCT during sleep stage N2 (b) in patient #13.

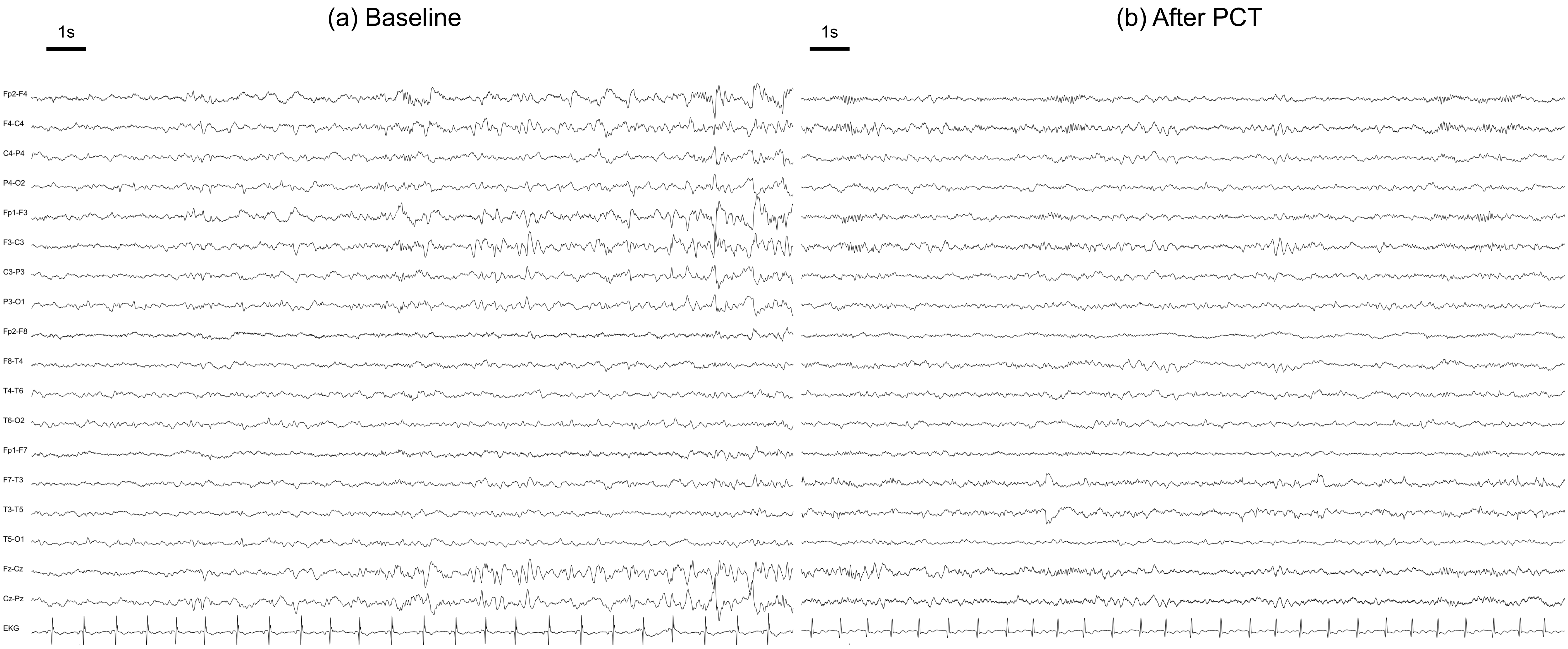

EEG recording with 19 channels in bipolar montage (15μV/mm, LF: 0.5, HF:50, 20s/page) during sleep stage N2 at baseline (a) and after 10 cycles of PCT during sleep stage N2 (b) in patient #14.

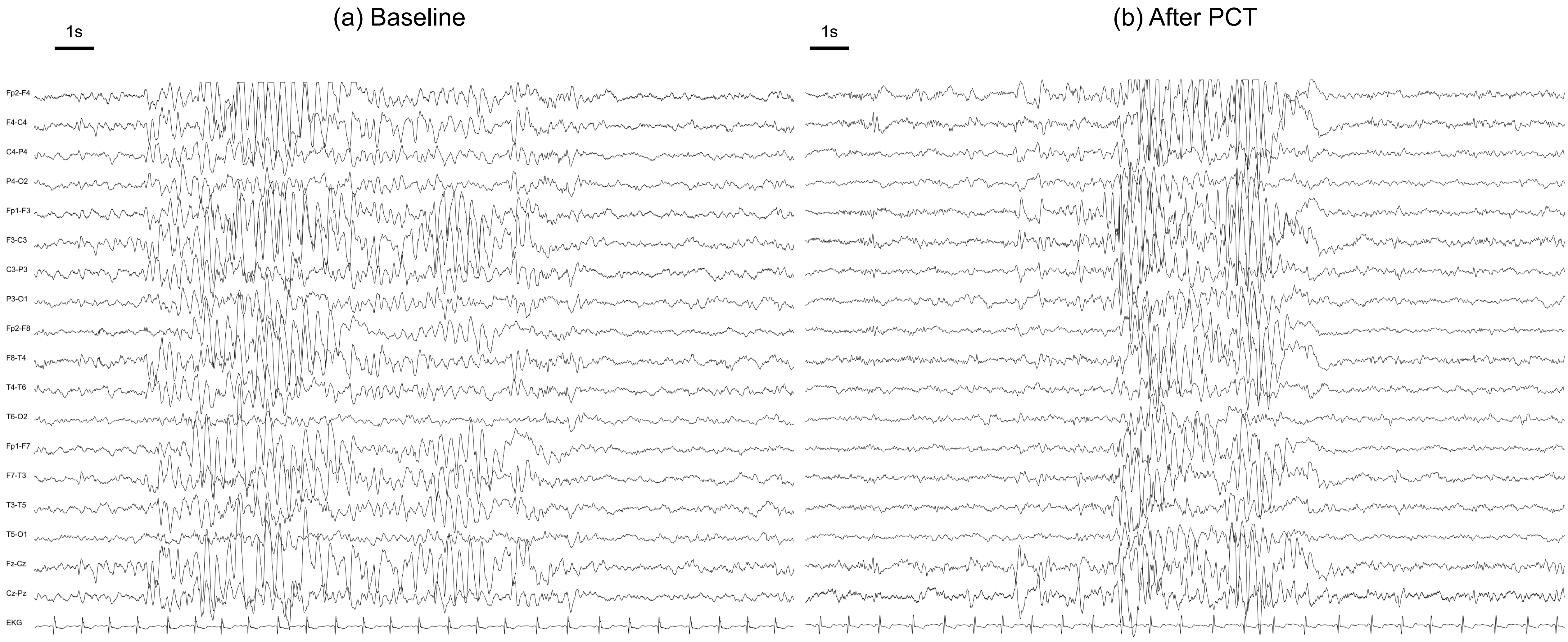

EEG recording with 19 channels in bipolar montage (20μV/mm, LF: 0.5, HF:35, 20s/page) during sleep stage N2 at baseline (a) and after 8 cycles of PCT during sleep stage N2 (b) in patient #15.

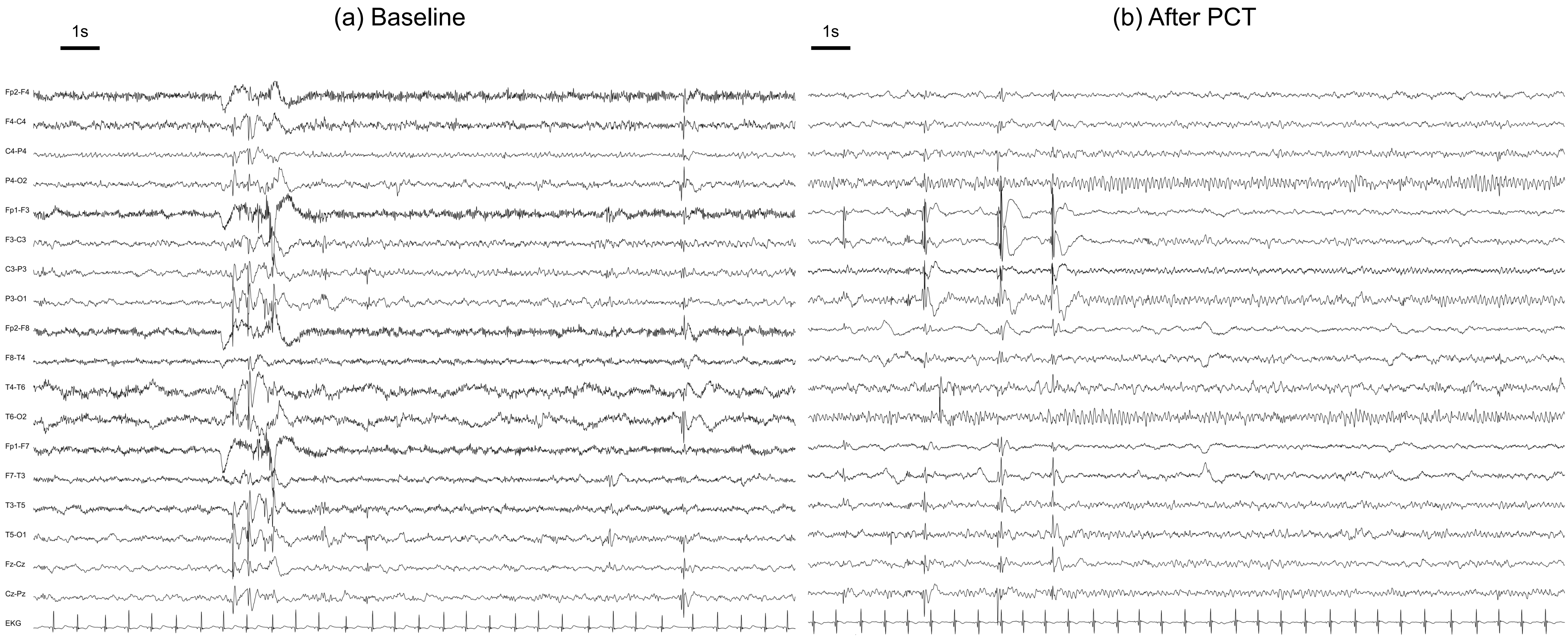

EEG recording with 19 channels in bipolar montage (30μV/mm, LF: 0.5, HF:70, 20s/page) during wakefulness at baseline (a) and after 8 cycles of PCT during wakefulness (b) in patient #16.

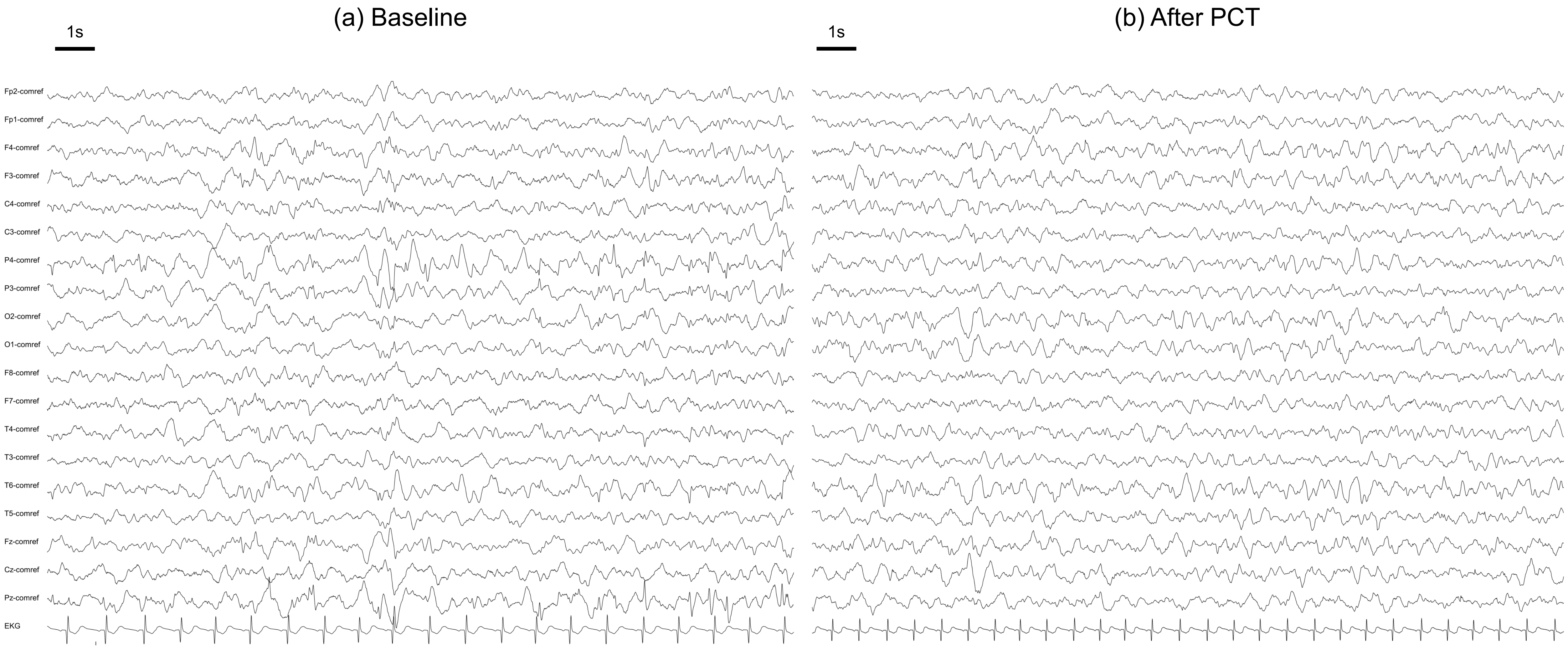

EEG recording with 19 channels in common average montage (20µV/mm, LF: 0.5, HF:35, 20s/page) during sleep stage N3 at baseline (a) and after 14 cycles of PCT during sleep stage N3 (b) in patient #17.

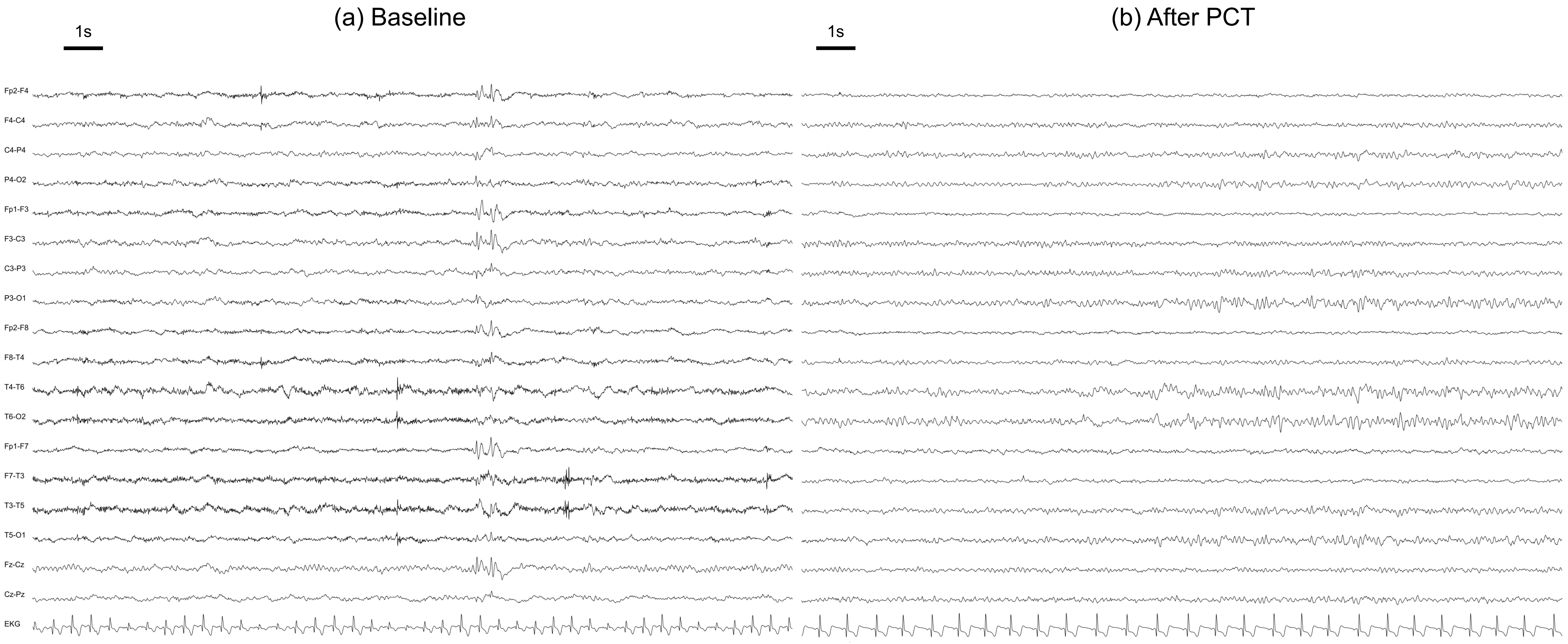

EEG recording with 19 channels in bipolar montage (20μV/mm, LF: 0.5, HF:70, 20s/page) during wakefulness at baseline (a) and after 4 cycles of PCT during wakefulness (b) in patient #18.

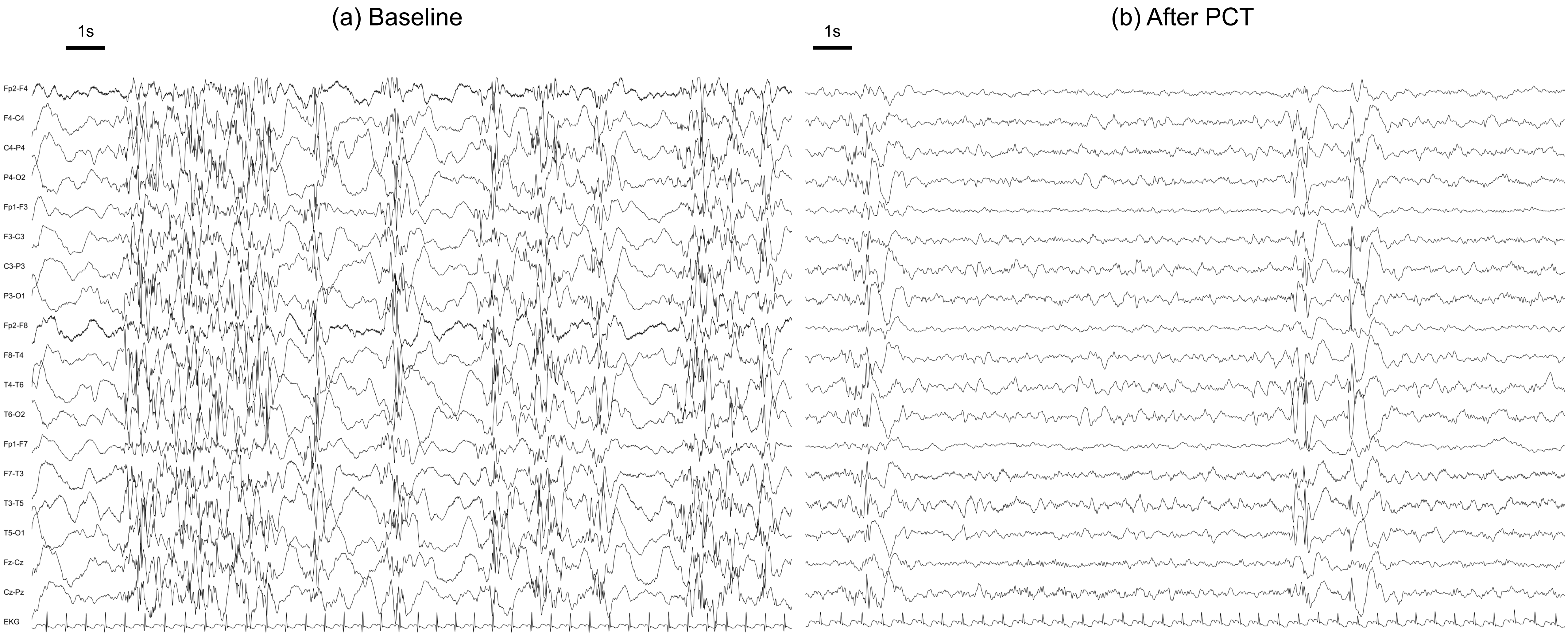

EEG recording with 19 channels in bipolar montage (20µV/mm, LF: 0.5, HF:70, 20s/page) during NREM sleep at baseline (a) and after 10 cycles of PCT during sleep stage N1 (b) in patient #19.

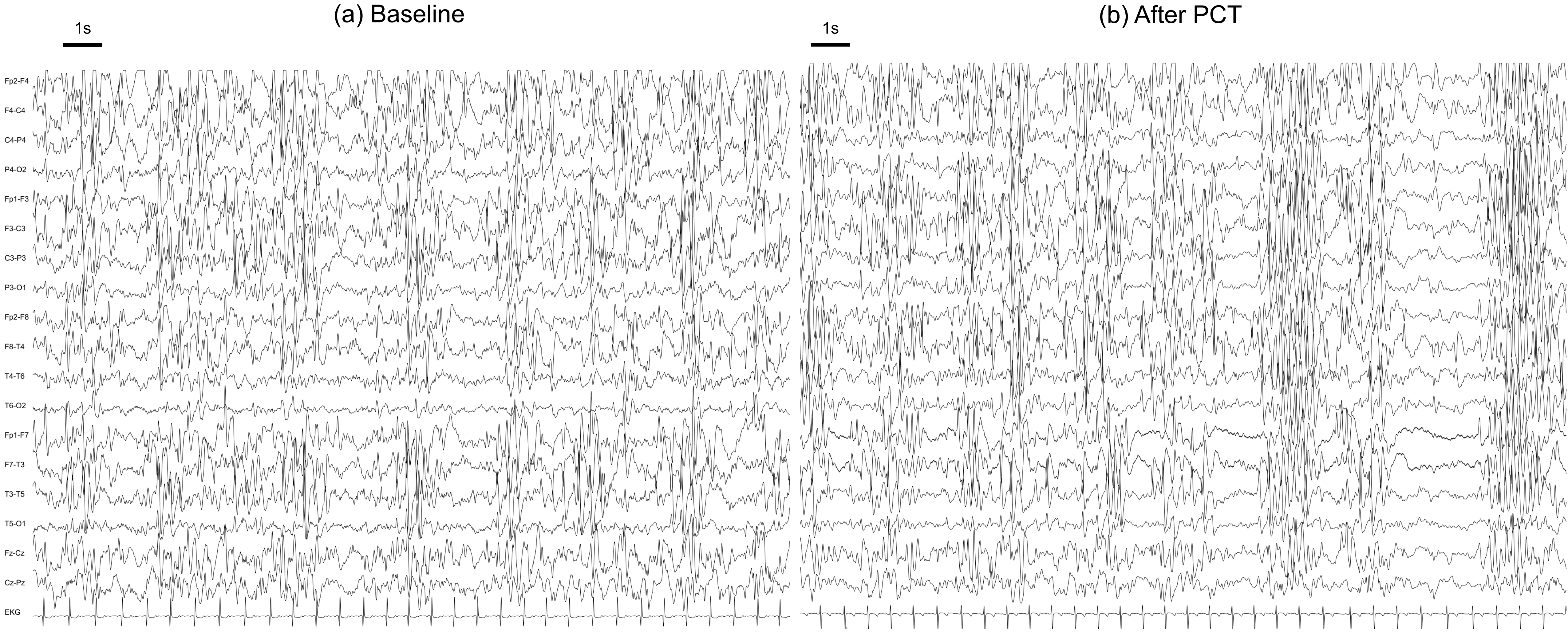

EEG recording with 19 channels in bipolar montage (30μV/mm, LF: 0.5, HF:70, 20s/page) during NREM sleep at baseline (a) and after 8 cycles of PCT during NREM sleep (b) in patient #20.

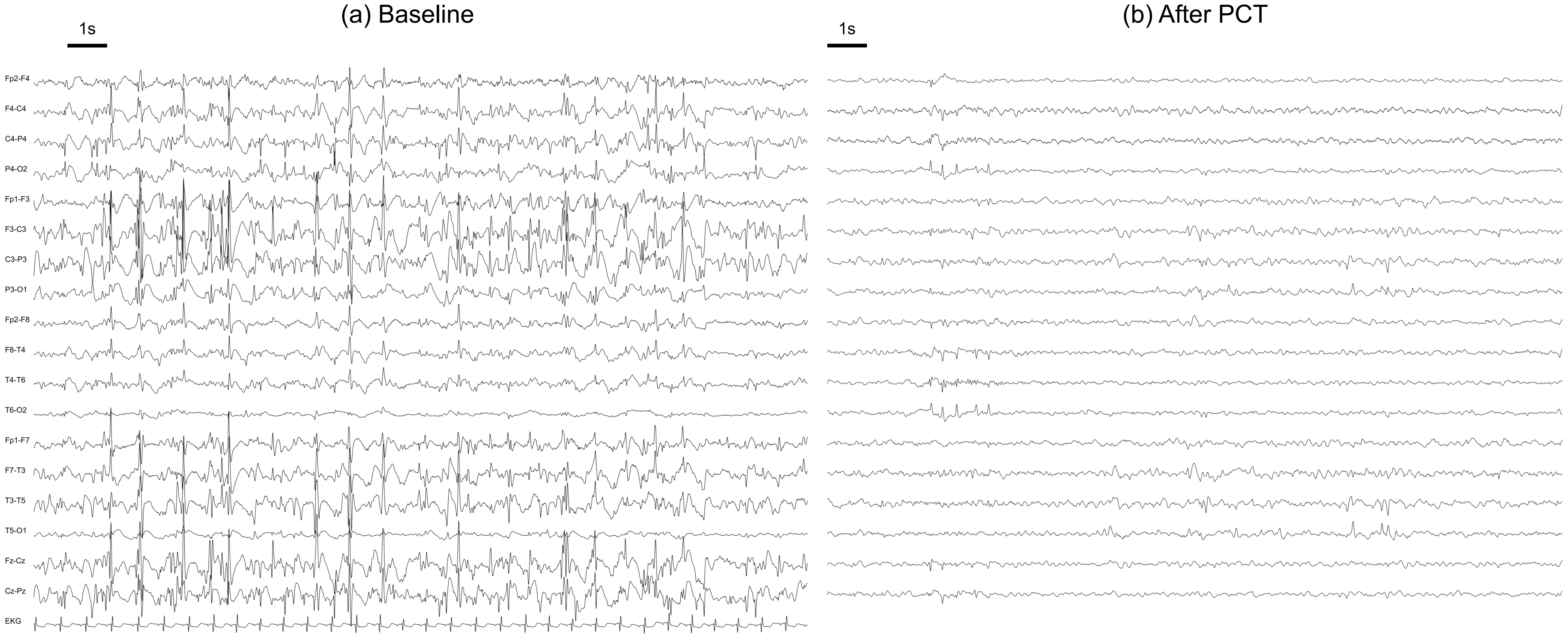

EEG recording with 19 channels in bipolar montage (20µV/mm, LF: 0.5, HF:70, 20s/page) during sleep stage N1 at baseline (a) and after 8 cycles of PCT during sleep stage N1 (EKG with artifacts) (b) in patient #21.

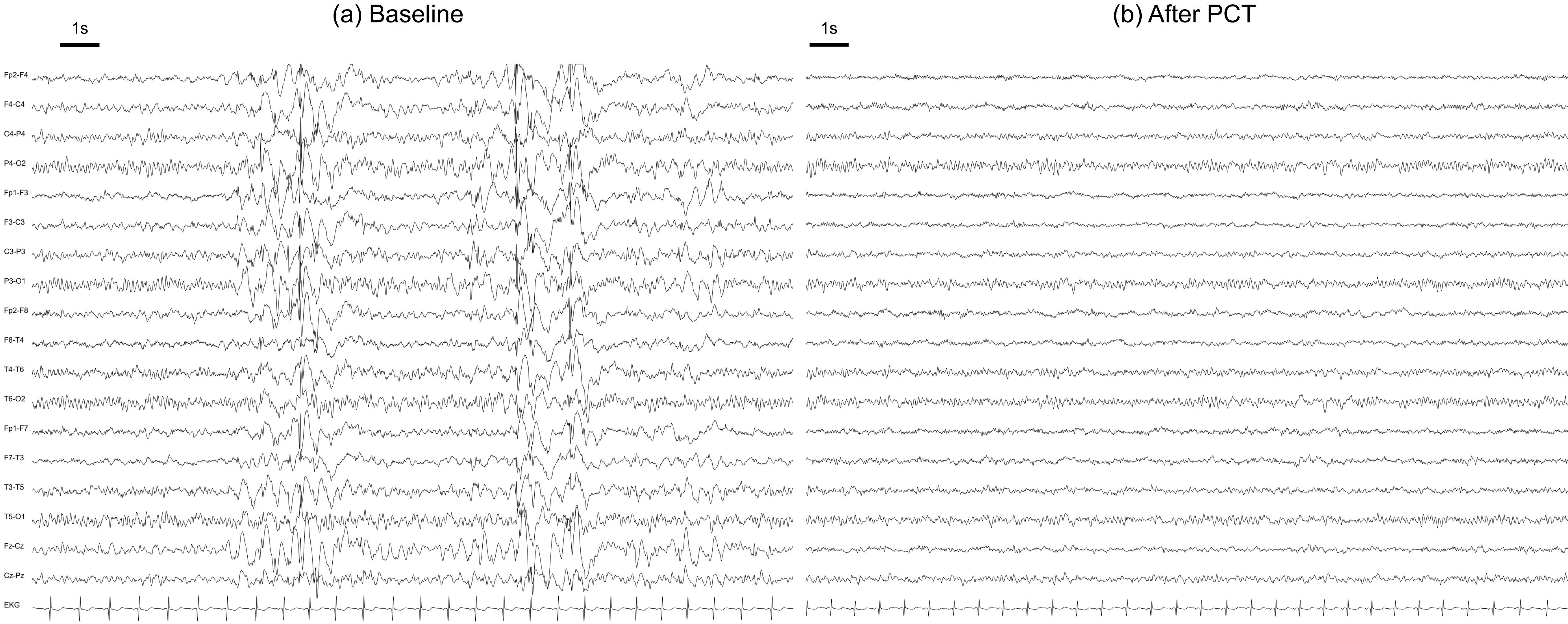

EEG recording with 19 channels in bipolar montage (20μV/mm, LF: 0.5, HF:70, 20s/page) during wakefulness at baseline (a) and after 6 cycles of PCT during wakefulness (b) in patient #22.

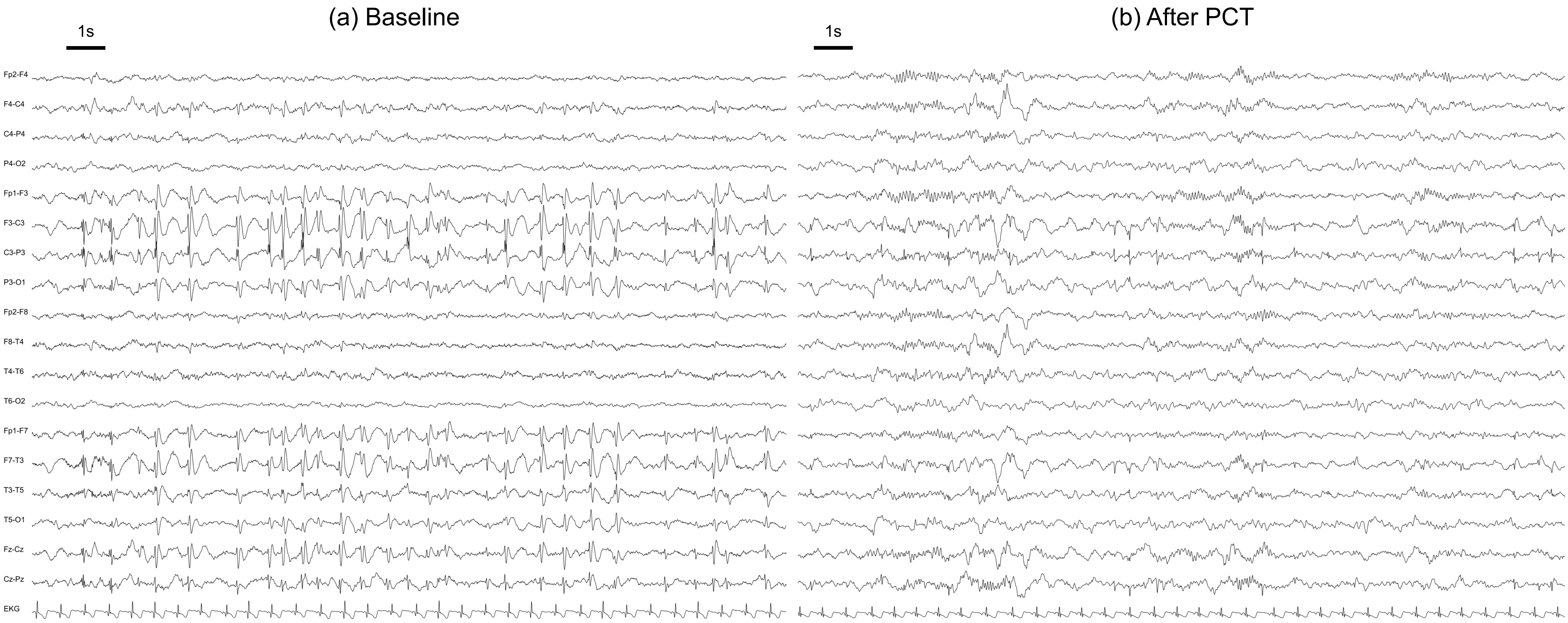

EEG recording with 19 channels in bipolar montage (20μV/mm, LF: 0.5, HF:70, 20s/page) during NREM sleep at baseline (a) and after 6 cycles of PCT during sleep stage N2 (b) in patient #23.

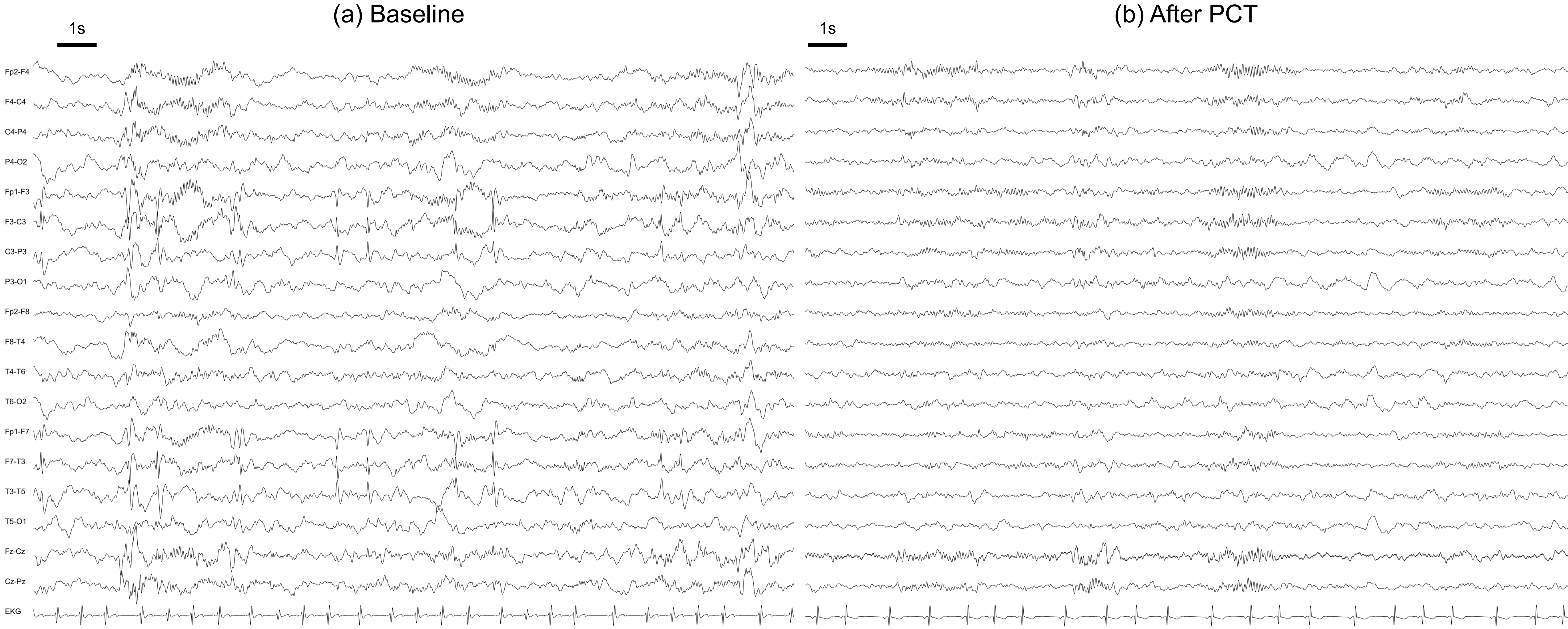

EEG recording with 19 channels in bipolar montage (20μV/mm, LF: 0.5, HF:70, 20s/page) during sleep stage N2 at baseline (a) and after 8 cycles of PCT during sleep stage N2 (b) in patient #24.
